# Hexacosenoyl-CoA is the most abundant very long-chain acyl-CoA in ATP binding cassette transporter D1-deficient cells[S]

**DOI:** 10.1194/jlr.P119000325

**Published:** 2020-02-19

**Authors:** Kotaro Hama, Yuko Fujiwara, Shigeo Takashima, Yasuhiro Hayashi, Atsushi Yamashita, Nobuyuki Shimozawa, Kazuaki Yokoyama

**Affiliations:** Faculty of Pharma-Science,* Teikyo University, Itabashi-ku, Tokyo 173-8605, Japan; Division of Genomics Research,† Life Science Research Center, Gifu University, Gifu 501-1193, Japan

**Keywords:** fatty acid, fatty acid/metabolism, lipids, lipidomics, peroxisomes, very long-chain fatty acid, X-linked adrenoleukodystrophy, hexacosenoyl (26:1)-coenzyme A, inherited metabolic disorder, adenosine 5′-triphosphate

## Abstract

X-linked adrenoleukodystrophy (X-ALD) is an inherited disorder caused by deleterious mutations in the *ABCD1* gene. The ABCD1 protein transports very long-chain FAs (VLCFAs) from the cytosol into the peroxisome where the VLCFAs are degraded through β-oxidation. ABCD1 dysfunction leads to VLCFA accumulation in individuals with X-ALD. FAs are activated by esterification to CoA before metabolic utilization. However, the intracellular pools and metabolic profiles of individual acyl-CoA esters have not been fully analyzed. In this study, we profiled the acyl-CoA species in fibroblasts from X-ALD patients and in ABCD1-deficient HeLa cells. We found that hexacosenoyl (26:1)-CoA, but not hexacosanoyl (26:0)-CoA, was the most abundantly concentrated among the VLCFA-CoA species in these cells. We also show that 26:1-CoA is mainly synthesized from oleoyl-CoA, and the metabolic turnover rate of 26:1-CoA was almost identical to that of oleoyl-CoA in both WT and ABCD1-deficient HeLa cells. The findings of our study provide precise quantitative and metabolic information of each acyl-CoA species in living cells. Our results suggest that VLCFA is endogenously synthesized as VLCFA-CoA through a FA elongation pathway and is then efficiently converted to other metabolites, such as phospholipids, in the absence of ABCD1.

Very long-chain FAs (VLCFAs) with no less than 24 carbons are endogenously synthesized through a FA elongation process (1). VLCFAs accumulate in several tissues, such as the skin and testes, but are maintained at a lower level in other tissues and in the plasma (2). VLCFAs are transported by ABCD1 into the peroxisome where they are degraded by β-oxidation. *ABCD1* is the causative gene of X-linked adrenoleukodystrophy (X-ALD), an inherited disorder that displays wide phenotypic variability including childhood-onset cerebral adrenoleukodystrophy (CCALD) and adrenomyeloneuropathy (AMN) (3). X-ALD is the most common inherited peroxisomal disorder with a prevalence of approximately 1/20,000 births, and most of those affected are males (3). Hematopoietic stem cell transplantation has been shown to provide long-term disease stabilization and survival (4, 5) and is thus one of the more promising treatment options for X-ALD. However, it remains necessary to develop drugs that can attenuate the progression of X-ALD in a patient-friendly therapeutic approach.

VLCFA levels are significantly elevated in all male X-ALD patients, but the extent thereof is not correlated with the severity or onset of clinical phenotypes (6). Thus, it remains unknown how VLCFA accumulation contributes to the progression of X-ALD, although recent observations propose that aberrant VLCFA metabolism may be associated with a proinflammatory state leading to the progression of X-ALD (7). Most VLCFAs are not present in the free form but are incorporated into complex lipids such as phospholipids (PLs) in humans (3) and mice (8). Total VLCFA contents have been profiled by gas chromatography-MS (9, 10), and recent progress in LC-MS analysis with a comprehensive lipidomic approach has revealed the endogenous form of VLCFAs in X-ALD patients to comprise part of PLs, glycolipids, and neutral lipids (11–13).

Fatty acyl-CoA (acyl-CoA) is an active form and serves as a metabolic intermediate of FAs (14). Acyl-CoA is composed of both a hydrophobic fatty acyl moiety and a hydrophobic CoA joined with a thioester linkage. This amphiphilic property hampers the efficient separation of individual acyl-CoA species when using conventional hydrophobic or hydrophilic interaction chromatography, and thus, the intracellular pool of each acyl-CoA ester has not yet been fully analyzed. In this study, we developed a LC-MS-based method to comprehensively profile the quantities and rates of metabolism of each acyl-CoA species in ABCD1-deficient cells.

## EXPERIMENTAL PROCEDURES

### Ethics

This study abides by the Declaration of Helsinki Principles, and the research protocol was approved by the Ethics Committee of Teikyo University (#12-078-6). Primary human fibroblasts were established from skin biopsy samples of four CCALD, three AMN, and five control patients. Informed consent was obtained from the human subjects or their representatives.

### Reagents

Phosphatidylcholine (PC) D_31_-16:0/18:1, phosphatidylethanolamine (PE) D_31_-16:0/18:1, and SM d18:1/D_31_-16:0 were purchased from Avanti Polar Lipids, Inc. (Alabaster, AL). All chemicals used in the mobile phases were purchased from Fujifilm Wako Pure Chemical Corporation (Osaka, Japan). Deuterium-labeled (12,12,13,13-D_4_) hexacosanoic acid (FA D_4_-26:0) and deuterium-labeled (9,10-D_2_) oleic acid (FA D_2_-18:1) were purchased from Cambridge Isotope Laboratories, Inc. (Andover, MA). Methyl-β-cyclodextrin (MβCD) was obtained from Sigma-Aldrich, Inc. (St. Louis, MO). FA D_4_-26:0 and FA D_2_-18:1 were dissolved in CHCl_3_/methanol (1:1 by volume, 3 mM each), and MβCD was dissolved in water (50 mM) and stored at −20°C. Deuterium-labeled palmitic acid (FA D_31_-16:0) and CoA were purchased from Santa Cruz Biotechnology (Dallas, TX) and the Oriental Yeast Co., Ltd. (Kyoto, Japan), respectively. Oxalyl chloride was purchased from Tokyo Chemical Industry (Tokyo, Japan). Other organic solvents used in the synthesis of FA D_31_-16:0 were purchased from Fujifilm Wako Pure Chemical Corporation.

### Synthesis of D_31_-palmitoyl-CoA

Deuterium-labeled palmitoyl-CoA (D_31_-16:0-CoA) was synthesized as described previously with slight modifications (15). Briefly, 2.5 mg of FA D_31_-16:0 and 2 ml of oxalyl chloride were mixed by stirring overnight at room temperature in a 10 ml ground-glass test tube to yield acyl chlorides. The excess oxalyl chloride was removed with nitrogen gas, and 2 ml of oxalyl chloride were added into the same test tube. After mixing for 1 h at room temperature, the excess oxalyl chloride was removed completely with nitrogen gas. The oil residue of FA chloride was dissolved in 0.5 ml of freshly distilled tetrahydrofuran (THF) and added into a CoA solution [10 mg of CoA dissolved in 2.3 ml of THF containing 0.1 M of Tris-HCl (pH 7.4) in a 2:1 ratio by volume] in a 10 ml screw-capped test tube. The acid chloride solution was added slowly to maintain the pH at ∼8 with the use of 1 M NaOH. Once all of the acid chloride solution had been added, the reaction mixture was adjusted to ∼pH 4 with 10% HClO_4_, and the THF was removed with nitrogen gas. Then, 1 ml of double-distilled water and 0.3 ml of 10% HClO_4_ were added into the reaction mixture, and the resulting white precipitate was collected by centrifugation (2,150 *g* for 10 min) at 4°C. The precipitate was washed with 4 ml of diethylether-petroleum ether (1:1 by volume), and the residue was dissolved in 100 μl of isopropanol followed by purification with HPLC. An Inertsil SIL-100A column (7.6 mm i.d. × 250 mm, particle size 5.0 μm; GL Science, Tokyo, Japan) was used at room temperature. The mobile phases were methanol/water (1:1 by volume) supplemented with 5 mM ammonium formate and 0.032% NH_4_OH. The flow rate was 1.5 ml/min. Each eluent fraction was diluted with methanol and directly infused into the mass spectrometer (Thermo LXQ; Thermo Fisher Scientific, Waltham, MA) to obtain MS/MS spectra. Fractions containing pure D_31_-16:0-CoA were combined and evaporated to dryness with the EZ-2 centrifugal evaporator (Genevac, Ipswich, UK). The obtained D_31_-16:0-CoA was reconstituted with methanol and stored at −20°C.

### Sample preparation

Sample preparation for acyl-CoA analysis was conducted as reported previously (16). Cell layers in the 100 mm culture dish were washed twice with PBS, scraped from the dishes, and collected by centrifugation (1,000 *g* for 5 min) at 4°C. Cell pellets were homogenized in 0.9 ml of acetonitrile/isopropanol (3:1 by volume). Homogenate protein concentrations were determined with a BCA protein assay kit (Thermo Fisher Scientific). Cell homogenates (approximately 0.5–1 mg protein) were spiked with 100 pmol of D_31_-16:0-CoA as the internal standard (IS) and 300 μl of 0.1 M KH_2_PO_4_ (pH 6.7) were added. After centrifugation (19,120 *g* for 5 min) at 4°C, each supernatant was mixed with 300 μl of acetic acid and loaded onto a 2-(2-pyridyl)ethyl silica gel column (Sigma-Aldrich) preconditioned with 1 ml of wash buffer (acetonitrile/isopropanol/water/acetic acid at 9:3:4:4 by volume). The loaded column was washed once with the wash buffer followed by the application of 1 ml of elution buffer (5 mM ammonium formate in a water/isopropanol mixture of 1:4 by volume). The eluent was evaporated completely with the EZ-2, and the resulting precipitate was reconstituted with 0.1 ml of methanol followed by filtration with a YMC Duo-Filter (4 mm i.d., pore size 0.2 μm; YMC Co., Ltd., Kyoto, Japan). Samples were stored at −20°C until analysis. For PL analysis, cell pellets were homogenized with 1.0 ml of methanol and the total lipid fraction was extracted as previously described (17).

### LC-MS/MS analysis

Quantitation of each acyl-CoA species was conducted with a QTRAP 4500 (Sciex, Framingham, MA) linked to a Nexera XR HPLC system (Shimadzu Corp., Kyoto, Japan). A Capcell Pak C_8_ UG120 column (1.5 mm i.d. × 35 mm, particle size 5.0 μm; Shiseido Co., Ltd., Tokyo, Japan) was used at 40°C. The mobile phases were 5 mM ammonium formate in water (pH 9.0) (mobile phase A) and 5 mM ammonium formate in a water/isopropanol solution (5:95 by volume; pH 9.0) (mobile phase B). The programmed solvent gradient consisted of solvents A/B at a 60/40 ratio for 2 min, programmed linear increments to 0/100 over 13 min, after which it was held at 0/100 for 2 min, and then linear increments to 60/40 over 1 min, after which it was held at 60/40 for 2 min. The flow rate of the mobile phase was 200 μl/min, and the volume of the injected samples was 5 μl. Multiple reaction monitoring (MRM) transitions were constructed to cover acyl-CoA species with 12–32 carbons and zero to six double bonds present in the acyl moieties. Each MRM transition was constructed by selecting protonated molecules ([M+H]^+^) and fragmented molecules ([M−C_10_H_15_N_5_O_13_P_3_]^+^) corresponding to the loss of a neutral fragment of a 3′-phosphoadenosine diphosphate as the precursor and product ions, respectively. The time period for data collection was 10 ms per cycle for each MRM transition. The following conditions were used for positive ion MRM: ion spray voltage, 5,500 V; temperature (TEM), 300°C; curtain gas (CUR), 40 arbitrary units (A.U.); collision gas (CAD), 9 A.U.; ion nebulizer gas (GS1), 40 A.U.; auxiliary gas (GS2), 80 A.U.; quadrupole mass filter (Q1) and Q3 linear ion trap (Q3/LIT) resolution, “unit”; declustering potential (DP), 1 V; entrance potential (EP), 10 V; collision energy (CE), 52.5 V; and collision cell exit potential, 12 V. Nitrogen was used as the nebulizer, curtain, and collision gas. Analyst software and MultiQuant software (Sciex) were used for data acquisition and processing. For structural analysis of VLCFA-CoA species, each acyl-CoA species was separated by HPLC, and the product ion spectra of VLCFA-CoA were obtained in the negative ion mode employing the MS^3^ scan to increase the signal-to-noise ratio of the product ion. The structure of each acyl-CoA species was assigned by the detection of the product ions corresponding to [M–phosphate–adenosine monophosphate]^−^ (*m/z* = M–348) and [adenosine diphosphate–H_2_O]^−^ (*m/z* = 408). Quantitative and structural analyses of the PL species were conducted with the LC-MS/MS and LC-MS^3^ method, respectively (8, 18).

### Method validation

Sample solutions for a spiked calibration curve were prepared as follows. A 100 μM stock solution of 17:0-CoA (Sigma-Aldrich) in methanol was prepared as the standard and this was diluted further with methanol to prepare standard solutions of 0.05, 0.1, 0.5, 1, 5, 10, 50, and 100 μM. A 10 μM stock solution of D_31_-16:0-CoA in methanol was prepared as the IS. Then, 50 μl of IS solution and each diluted standard solution were placed into a 2.0 ml siliconized plastic tube and mixed with 0.6 ml of HeLa cell homogenate containing 0.6 mg protein in acetonitrile/isopropanol (3:1 by volume), followed by the addition of 200 μl of 0.1 M KH_2_PO_4_ (pH 6.7). Next, the supernatant was collected by centrifugation (19,120 *g* for 5 min) at 4°C and mixed with 200 μl of acetic acid. Each sample was loaded onto a 2-(2-pyridyl)ethyl silica gel column (Sigma-Aldrich) preconditioned with 1 ml of wash buffer (acetonitrile/isopropanol/water/acetic acid at a ratio of 9:3:4:4 by volume), and processed using a procedure identical with that previously described above (Sample preparation section). For validation of the method, three samples containing 0.05, 1, and 10 pmol of standard per injection were analyzed for quality control (QC) purposes. To generate a linear regression curve, 1/*x*^2^ was used as a weighting factor (19). Accuracy was calculated as: [(observed concentration − endogenous concentration)/nominal concentration − 1] × 100 (%) and the coefficient of variation was evaluated to determine measurement precision.

### Cell lines and cell culture

HeLa cells were obtained from the cell bank of the Riken Bioresource Center (Ibaraki, Japan). Three clonal WT HeLa cell lines were generated by the limiting dilution method, and one of these clones was used to generate ABCD1-KO HeLa cell lines. Human skin fibroblasts and HeLa cells were cultured in minimum essential medium (Sigma-Aldrich) supplemented with 10% FBS (Biowest, Nuaillé, France), 2 mM l-glutamine (Thermo Fisher Scientific), 100 U ml^−1^ penicillin, and 100 μg ml^−1^ streptomycin (Sigma-Aldrich). Prior to metabolic labeling experiments, FA D_2_-18:1 or FA D_4_-26:0 was mixed with MβCD to form the FA/MβCD complexes as described previously (20). WT and ABCD1-KO HeLa cells were seeded (2.0 × 10^6^ cells per 10 cm culture dish) and cultured overnight in medium containing 10% FBS. Then, the cells were treated with 30 μM of the FA D_2_-18:1/MβCD or FA D_4_-26:0/MβCD complex and harvested at the indicated time points after treatment. For the pulse-chase experiments, wild-type and ABCD1-KO HeLa cells were seeded (2.0 × 10^6^ cells per 10 cm culture dish) and cultured overnight in medium containing 10% FBS. Then, the cells were treated with 30 μM of FA D_2_-18:1/MβCD complex for 1 h, following which the medium was replaced with fresh culture medium containing 10% FBS. The cells were then harvested at the indicated time points.

### Generation of ABCD1-KO HeLa cells with the CRISPR/Cas9 system

To establish the ABCD1-KO HeLa cell lines, we designed two guide RNAs for the human *ABCD1* gene using the CRISPR design tool (http://crispor.tefor.net/). Plasmid construction was performed as previously described (21). Briefly, oligonucleotide pairs for the *ABCD1* gRNA 1 (5′-CACCGTGGGCTCCATAGGCCGCGA-3′ and 5′-AAACTCGCGGCCTATGGAGCCCAC-3′) and *ABCD1* gRNA 2 (5′-CACCGCCAGGCACTGGCGCACCAA-3′ and 5′-AAACTTGGTGCGCCAGTGCCTGGC-3′) were cloned into the pSpCas9(BB)-2A-GFP (PX458) vector. The constructs were transfected into HeLa cells using Lipofectamine 2000 (Thermo Fisher Scientific) according WTthe manufacturer’s instructions. EGFP-positive cells were collected with a FACSAria III (BD Biosciences, Franklin Lakes, NJ) at 3 days after transfection, and clonal populations were obtained by limiting dilution. ABCD1 deficiency was screened with a PCR-restriction enzyme (PCR-RE) assay. The oligonucleotide pairs used in the PCR-RE assay for both gRNA target sequences were 5′- TCTCCAGGCCCCGGCCCTG-3′ and 5′-CAGCTGCCAGCCAAAAGCCCGC-3′. The restriction enzymes used in the PCR-RE assay were *Bgl*I and *Sty*I for gRNA1 and -2, respectively. The obtained PCR products were subcloned into a T-vector (pMD20; Takara Bio, Inc., Shiga, Japan) and sequenced. Three clonal ABCD1-KO cell lines were generated and termed ABCD1-KO (#1), ABCD1-KO (#2), and ABCD1-KO (#3).

### Statistical methods

Statistical analysis was performed with either one-way ANOVA followed by the Dunnett T3 or Tukey post hoc test or with the Student’s *t*-test or Mann-Whitney *U* test. Differences were considered to be significant if the *P*-value was <0.05. All statistical analyses were conducted with IBM SPSS Statistics version 23 (IBM, Armonk, NY). Spectral data were plotted with MjoGraph software (Ochiai Laboratory, Yokohama National University, Japan).

## RESULTS

### Development of an LC-MS method for acyl-CoA analysis

**Fig. 1.**
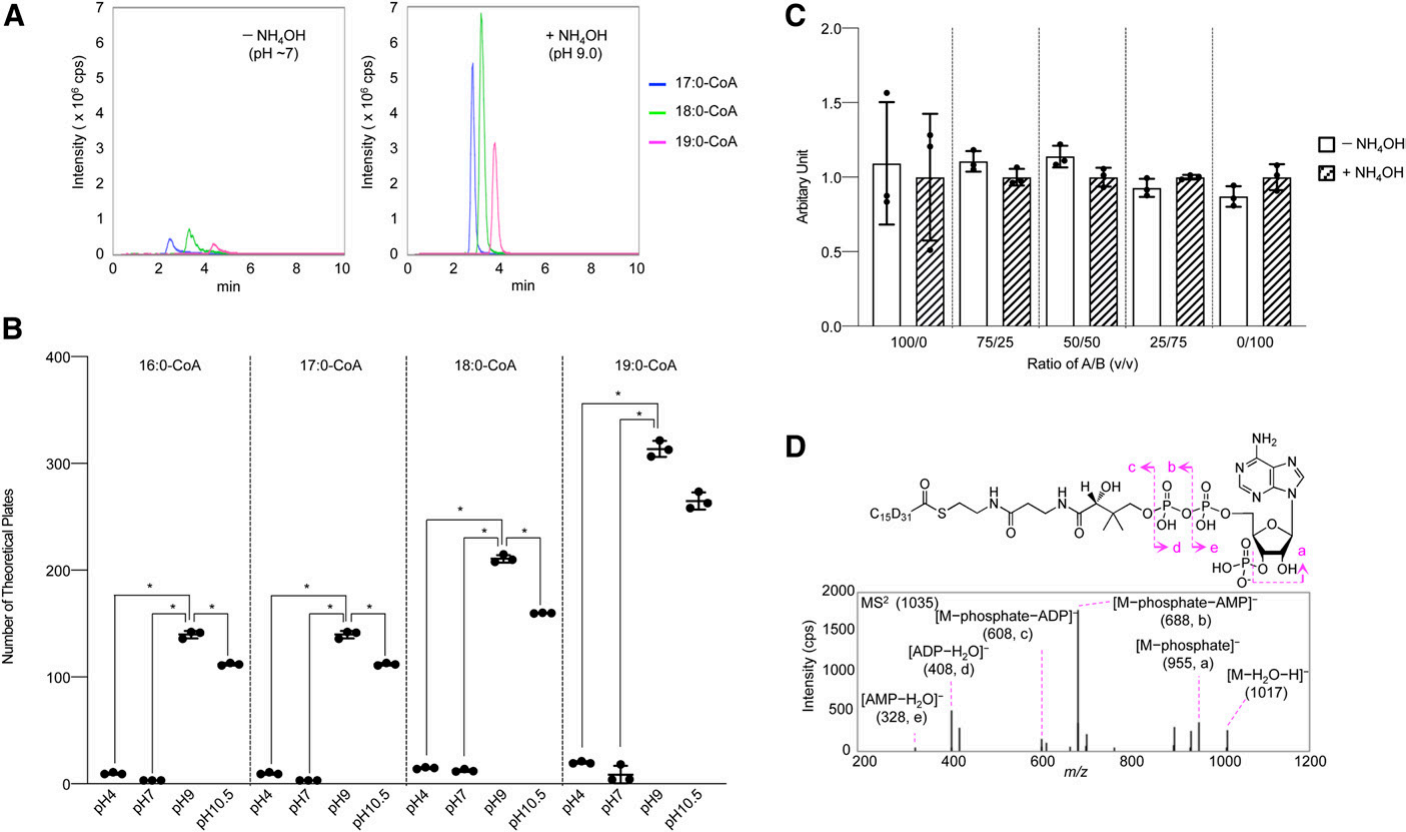
Development of an LC-MS method for acyl-CoA analysis. A: The effect of the mobile phase pH on the separation and sensitivity of each acyl-CoA species. A mixture containing 50 pmol of 17:0-, 18.0-, or 19.0-CoA was analyzed by LC-MS using mobile phases adjusted to either ∼pH 7 or pH 9.0 with ammonium hydroxide. B: The effect of mobile phase pH on separation performance for acyl-CoA analysis. The number of theoretical plates for all four acyl-CoA species (16:0-, 17:0-, 18:0-, and 19:0-CoA) was highest when the mobile phases were adjusted to pH 9.0. C: The stability of acyl-CoA under alkaline conditions. A solution of 17:0-CoA was preincubated for 1 h with mobile phases adjusted to (pH 9.0) or without ammonium hydroxide (∼pH 7) and analyzed by LC-MS using mobile phases adjusted to pH 9.0. No significant difference was observed in the sensitivity of 17:0-CoA detection in the pretreated samples irrespective of the ratio of mobile phase A (water) to B (isopropanol) used during preincubation. Data represent the mean ± SD, n = 3. D: The MS/MS (MS^2^) spectra of chemically synthesized D_31_-16:0-CoA corresponding to *m/z* = 1,035 ([M−H]^–^). Spectra for both hydrophobic ([M−phosphate]^–^, [M−phosphate−adenosine monophosphate]^–^, and [adenosine diphosphate-water]^–^ and [adenosine diphosphate−water]^–^) were observed. Statistical analysis was performed with one-way ANOVA followed by the Tukey post hoc test in B and the Student’s *t*-test in C. *P < 0.05 for comparisons between pH 4.0, pH 6.5, pH 9.0, and pH 10.5 (B) or between −NH_4_OH and +NH_4_OH (C).

Free FAs are linked to CoA by acyl-CoA synthetase and pooled as intermediates of metabolic processes. To explore how ABCD1 deficiency influences the metabolism of each FA, we developed an LC-MS method for acyl-CoA analysis and used it to profile intracellular acyl-CoA species. Due to the amphiphilic property of acyl-CoA, ion-pairing reagents have been used to separate each acyl-CoA species by the reversed-phase HPLC methods developed thus far (22). However, it is desirable to avoid the usage of ion-pairing reagents because it is difficult to remove these reagents completely from the HPLC column after use. Among the LC-MS methods developed to date for acyl-CoA analysis, we referred to the method reported by Magnes et al. (23) for further development in which ion-pairing reagents were not used to separate each acyl-CoA species. In their study, C_18_ reversed-phase columns were used at high pH (pH 10.5) with an ammonium hydroxide and acetonitrile gradient. Here, we utilized C_8_ reversed-phase columns with an ammonium hydroxide and isopropanol gradient to efficiently elute the VLCFA-CoA species. We found that each acyl-CoA species was separated with high sensitivity when both mobile phases were adjusted to pH 9.0 by ammonium hydroxide (**Fig. 1A**). We also tested acidic (pH 4.0), weakly acidic (pH 6.5), and high pH (pH 10.5) conditions and found that the maximum number of theoretical plates for each acyl-CoA species was obtained when the mobile phases were adjusted to pH 9.0 (Fig. 1B). Because the thioester bond of acyl-CoA is sensitive to hydrolysis at high pH, we next examined whether the acyl-CoA species were degraded in the mobile phases at pH 9.0. We incubated 17:0-CoA with the mobile phases for 1 h before analysis with various ratios of water to isopropanol and either with or without ammonium hydroxide. We did not observe any significant effect of the pH of the mobile phases (Fig. 1C) and concluded that the acyl-CoA species were not degraded under the HPLC gradient conditions used in this study.

IS compounds are critical for LC-MS-based quantitation (24). Stable isotopically labeled analogs are desirable as IS compounds because these nonnatural compounds can be quantified without any signal overlap with natural compounds, and such standards can properly compensate for variability in sample extraction and LC-MS analysis. Therefore, we chemically synthesized D_31_-16:0-CoA and confirmed its structure by MS/MS analysis (Fig. 1D). We then validated our method for the quantitation of each acyl-CoA species. Two acyl-CoA species were used to construct a spiked calibration curve that was applicable to acyl-CoA species with cell homogenates as a biological matrix, namely 17:0-CoA and D_31_-16:0-CoA, which served as the standard compound and the IS, respectively. Spiked standard solutions were analyzed using a scheduled MRM mode and the linearity was examined over the range of 0.05–100 pmol per injection (**Table 1**). Accuracy and precision values for the 1 and 10 pmol per injection were within 15%, and these values for the 0.05 pmol per injection were identical with the lower limit of the quantitation range and were almost within 20% (Table 1). These results showed that 0.05–100 pmol of acyl-CoA species could be quantified with the present quantitative method.

**TABLE 1.** Calibration curve for the acyl-CoA species

			Linearity				Precision [CV(%)]*^a^*			Accuracy (%)		
Compound	Range (pmol)	Weight	Slope	Intercept	*r*^2^		QC-L*^b^*	QC-M*^b^*	QC-H*^b^*	QC-L*^b^*	QC-M*^b^*	QC-H*^b^*
17:0-CoA/(D_31_)-16:0-CoA	0.05–100	1/*x*^2^	0.061	−0.0024	0.991	Intra-day (n = 12)	10.6	6.3	3.7	9.5	5.2	5.8
						Inter-day (n = 4+4+4)	21.2	10.3	6.3	21.2	11.8	9.2

a Precision was calculated as the coefficient of variation (CV).

b Three samples with 0.050, 1.0, and 10 pmol of 17:0-CoA were mixed with 10 pmol of (D_31_)-16:0-CoA per injection and were analyzed as QC compounds QC-L (low), QC-M (middle), and QC-H (high), respectively.

### Quantitative analysis of acyl-CoA species in X-ALD fibroblasts

**Fig. 2.**
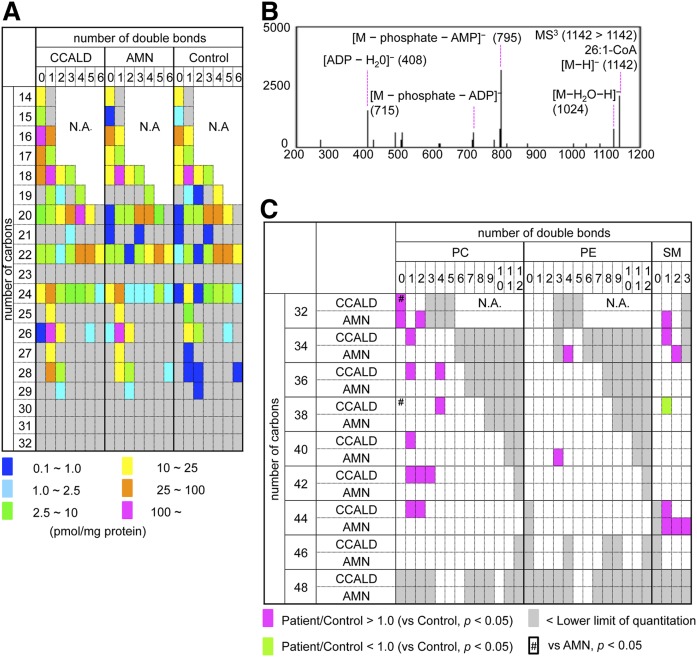
Quantity of each acyl-CoA species in the X-ALD fibroblasts. A: Each acyl-CoA species in fibroblasts from four CCALD, three AMN, and five control patients was quantified in the positive ion mode, and was classified according to the number of carbons and double bonds in the acyl moiety. The ratio of peak area for each acyl-CoA/D_31_-16:0-CoA was used to calculate the amount of each acyl-CoA species, and the mean quantity of each acyl-CoA species in the X-ALD fibroblasts is represented with a color key. The data are also summarized in Table 2. Acyl-CoA species observed to be present below the quantitation range are indicated in gray. B: The product ion spectra of 26:1-CoA corresponding to [M–H]^−^ (*m/z* = 1,142) in LC-MS^3^ analysis. Structural analysis was performed in the negative ion mode. C: Each PC, PE, and SM species present in quantities significantly higher (magenta) or lower (lime) in X-ALD fibroblast samples (CCALD or AMN) relative to the control fibroblasts was classified according to the number of carbons and double bonds in the two acyl moieties. The PL species present in quantities significantly altered in CCALD fibroblasts relative to the AMN fibroblasts are indicated as #. The PL species observed to be present in quantities below the quantitation range are indicated in gray. Acyl-CoA species for which the MRM channels were not designed are indicated as N.A. The quantity of each PL species in the X-ALD fibroblasts is listed in supplemental Table S1

Using the LC-MS method we developed for acyl-CoA analysis, we first profiled each acyl-CoA species in fibroblasts from four CCALD, three AMN, and five control patients. Among the 112 acyl-CoA species analyzed in the fibroblasts cultured with medium containing 10% FBS, we found 43, 42, and 44 species to be within the quantitative range in X-ALD fibroblasts from CCALD, AMN, and the control patients, respectively (**Fig. 2A**, **Table 2**). VLCFA-CoA species with no less than 24 carbons in their fatty acyl moiety were significantly accumulated in both CCALD and AMN fibroblast samples compared with the control, and hexacosenoyl-CoA (26:1-CoA) was the most and the second most abundant acyl-CoA species in both AMN and CCALD fibroblast samples, respectively (Fig. 2B, Table 2). It is also notable that the proportion of VLCFA-CoA species with one and two double bonds was significantly higher in both CCALD and AMN fibroblasts (Fig. 2A, Table 2). Accumulated acyl-CoA pools in cells are partially used for the synthesis of a variety of PL species (25). To examine possible correlations between the profiles of acyl-CoA species and PL species, we quantified each PL species in both CCALD and AMN fibroblasts. Among the PL species observed within the quantitative range, eleven PC species (PC 32:0, 34:1, 36:1, 36:4, 38:4, 40:1, 42:1, 42:2, 42:3, 44:1, and 44:2) and two SM species (SM 34:1 and 44:1) were present at significantly higher levels in CCALD fibroblasts as compared with control fibroblasts (Fig. 2C, supplemental Table S1). Notably, structural analysis by LC-MS^3^ revealed that five (24:0, 24:1, 24:2, 26:0 and 26:1) and two (26:0 and 26:1) fatty acyl moieties were mainly present as VLCFAs in six of the PC species (PC 40:1, 42:1, 42:2, 42:3, 44:1, and 44:2) and two of the SM species (SM 44:1 and 44:2), respectively (**Table 3**, supplemental Figure S1). Only one SM species (SM 38:1), which was identified as SM d18:1/20:0, was present at significantly lower levels in CCALD fibroblasts (Fig. 2C, supplemental Table S1, supplemental Fig. S1). In contrast, two PC species (PC 32:0 and 32:2), two PE species (PE 34:4 and 40:3), and five SM species (SM 32:1, 34:2, 44:1, 44:2, and 44:3) were present at a significantly higher level in AMN fibroblasts as compared with control fibroblasts (Fig. 2C, supplemental Table S1, supplemental Fig. S1). Significant difference was not observed in the amount of PC species with VLCFAs, such as PC 44:1 and 44:2, mainly due to the wide penetrance differences in the quantity of these PC species in three AMN fibroblasts (supplemental Table S1). These results show that acyl-CoA species with 26 carbons and zero to two double bonds in their acyl moieties are mainly accumulated as VLCFA-CoA pools and are possibly transferred into complex lipids, such as PC and SM. Interestingly, significant differences were observed in the amount of three acyl-CoA species (20:2-, 22:5-, and 24:5-CoA) and two PC species (PC 32:0 and PC 38:0) between CCALD and AMN fibroblasts (Fig. 2A, C; Tables 2, 3; supplemental Table S1). These results may reflect the difference in the metabolism of saturated and polyunsaturated long-chain FAs between CCALD and AMN fibroblasts.

**TABLE 2.** Quantification of the acyl-CoA species in the X-ALD fibroblasts

				CCALD (n = 4)		AMN (n = 3)		Control (n = 5)	
Signals	Q1 (*m/z*)	Q3 (*m/z*)	Retention Time (min)	Amount*^a^*	Percent	Amount*^a^*	Percent	Amount*^a^*	Percent
14:0-CoA	978	471	0.97	18.3	1.6	13.9	1.9	15.5	2.3
15:0-CoA	992	485	1.12	3.2	0.3	0.6	0.1	1.9	0.3
16:0-CoA	1,006	499	1.34	102.0	8.9	48.6	6.5	71.4	10.5
16:1-CoA	1,004	497	1.06	28.8	2.5	16.3	2.2	22.5	3.3
17:0-CoA	1,020	513	1.62	25.5	2.2	18.2	2.4	23.8	3.5
17:1-CoA	1,018	511	1.24	8.4	0.7	4.3	0.6	5.0	0.7
18:0-CoA	1,034	527	2.07	31.8	2.8	15.6	2.1	21.4	3.2
18:1-CoA	1,032	525	1.48	211.0	18.5	108.3	14.5	135.2	19.9
18:2-CoA	1,030	523	1.21	23.9	2.1	15.4	2.1	18.5	2.7
18:3-CoA	1,028	521	1.05	6.6*^b^*	0.6	2.7	0.4	3.7	0.5
19:1-CoA	1,046	539	1.79	3.3	0.3	n.q.	0.0	1.1	0.2
19:2-CoA	1,044	537	1.40	1.3	0.1	n.q.	0.0	0.8	0.1
19:4-CoA	1,040	533	1.30	9.4	0.8	8.8	1.2	12.5	1.8
20:0-CoA	1,062	555	3.56	3.2	0.3	0.4	0.1	0.9	0.1
20:1-CoA	1,060	553	2.25	9.9	0.9	5.5	0.7	5.9	0.9
20:2-CoA	1,058	551	1.64	12.5*^c^*	1.1	6.1	0.8	7.8	1.1
20:3-CoA	1,056	549	1.36	74.7	6.5	43.8	5.9	54.9	8.1
20:4-CoA	1,054	547	1.19	109.3	9.6	82.1	11.0	85.8	12.6
20:5-CoA	1,052	545	1.03	18.5	1.6	7.5	1.0	12.1	1.8
21:0-CoA	1,072	565	1.27	n.q.	0.0	0.6	0.1	1.0	0.1
21:3-CoA	1,066	559	1.66	1.8*^b^*	0.2	0.5	0.1	0.2	0.0
22:0-CoA	1,090	583	5.28	5.1*^b^*	0.4	4.2	0.6	1.5	0.2
22:1-CoA	1,088	581	3.75	8.0	0.7	6.9	0.9	6.3	0.9
22:2-CoA	1,086	579	2.67	1.4	0.1	0.4	0.0	0.6	0.1
22:3-CoA	1,084	577	2.00	7.8	0.7	3.7	0.5	6.7	1.0
22:4-CoA	1,082	575	1.62	50.0	4.4	18.0	2.4	31.6	4.6
22:5-CoA	1,080	573	1.35	64.3*^c^*	5.6	32.0	4.3	46.5	6.8
22:6-CoA	1,078	571	1.20	22.2	1.9	11.5	1.5	16.8	2.5
24:0-CoA	1,118	611	6.46	10.8*^b^*	0.9	16.7	2.2	0.9	0.1
24:1-CoA	1,116	609	5.32	33.6*^b^*	2.9	33.4*^b^*	4.5	12.9	1.9
24:2-CoA	1,114	607	4.40	2.7	0.2	1.3	0.2	0.8	0.1
24:3-CoA	1,112	605	3.21	3.7	0.3	1.8	0.2	3.2	0.5
24:4-CoA	1,110	603	2.40	5.3	0.5	1.6*^b^*	0.2	8.7	1.3
24:5-CoA	1,108	601	1.94	7.5*^c^*	0.7	3.6*^b^*	0.5	6.5	1.0
24:6-CoA	1,106	599	1.62	2.1	0.2	1.0	0.1	1.3	0.2
25:1-CoA	1,130	623	5.92	15.2	1.3	16.6	2.2	9.2	1.4
26:0-CoA	1,146	639	7.50	0.9	0.1	1.6	0.2	n.q.	0.0
26:1-CoA	1,144	637	6.44	132.1*^b^*	11.6	134.7*^d^*	18.1	17.9	2.6
26:2-CoA	1,142	635	5.79	15.3*^b^*	1.3	11.2	1.5	3.6	0.5
26:5-CoA	1,136	629	2.96	2.3	0.2	n.q.	0.0	1.6	0.2
27:1-CoA	1,158	651	6.99	11.7*^d^*	1.0	10.9*^d^*	1.5	0.2	0.0
28:1-CoA	1,172	665	7.49	26.0*^b^*	2.3	24.1*^b^*	3.2	0.1	0.0
28:2-CoA	1,170	663	6.72	7.2*^b^*	0.6	6.5	0.9	0.2	0.0
28:6-CoA	1,162	655	6.07	n.q.	0.0	1.1	0.2	0.2	0.0
29:2-CoA	1,184	677	6.52	1.6	0.1	1.3	0.2	0.5	0.1
Others	—	—	—	3.2	0.3	0.9	0.1	0.3	0.0
Total	—	—	—	1,143.4*^b^*	100.0	744.3	100.0	680.7	100.0
VLCFA-CoA (DB = 0)*^e^*				11.7	1.0	18.2	2.4	0.9	0.1
VLCFA-CoA (DB = 1)*^e^*				218.6*^b^*	19.1	219.8*^d^*	29.5	40.5	5.9
VLCFA-CoA (DB = 2)*^e^*				26.8*^b^*	2.3	20.3*^b^*	2.7	5.0	0.7
VLCFA-CoA (DB = 3)*^e^*				3.7	0.3	1.8	0.2	3.2	0.5
VLCFA-CoA (DB = 4)*^e^*				6.0	0.5	1.6*^b^*	0.2	8.7	1.3
VLCFA-CoA (DB = 5)*^e^*				9.8	0.9	3.6	0.5	8.1	1.2
VLCFA-CoA (DB = 6)*^e^*				2.8	0.2	2.3	0.3	1.9	0.3
Total of VLCFA-CoA				279.4*^b^*	24.4	267.5*^d^*	35.9	68.3	10.0

a Expressed as picomoles per milligram of protein. The ratio of peak area for each acyl-CoA/D_31_-16:0-CoA was used to calculate the amount of each acyl-CoA species. n.q., below the quantitation range.

b *P* < 0.05 versus control (Dunnett T3 post hoc test).

c *P* < 0.05 versus AMN (Dunnett T3 post hoc test).

d *P* < 0.01 versus control (Dunnett T3 post hoc test).

e The total amount of each VLCFA-CoA species that contain very long-chain fatty acyl moieties with the number of double bonds (DBs) as indicated.

**TABLE 3.** Structure of the PL species present in quantities significantly higher in the X-ALD fibroblasts

	Amount*^c^*										
Signals*^a^*,*^b^*	CCALD	AMN	Control	*^d^*	*sn*-1 FA (*m/z*)*^e^*	*sn*-2 FA (*m/z*)*^e^*	1-Acyl LPC (*m/z*)*^e^*	Molecular Species	*N-*FA (*m/z*)*^e^*	SPC (*m/z*)*^e^*	Molecular Species
PC 32:0	5,625*^f^*,*^g^*	3,939	3,741	778	18:0 (283)	14:0 (227)	LPC 18:0 (508)	PC 18:0/14:0	—	—	—
					16:0 (283)	16:0 (227)	LPC 16:0 (480)	PC 16:0/16:0	—	—	—
PC 32:2	883	767*^f^*	640	774	16:0 (255)	16:2 (251)	LPC 16:0 (480)	PC 16:0/16:2	—	—	—
					16:1 (253)	16:1 (253)	LPC 16:1 (478)	PC 16:1/16:1	—	—	—
					14:0 (227)	18:2 (279)	LPC 14:0 (452)	PC 14:0/18:2	—	—	—
PC 34:1	10,675*^h^*	8,289	6,469	804	16:0 (255)	18:1 (281)	LPC 16:0 (480)	PC 16:0/18:1	—	—	—
PC 36:1	8,808*^f^*	8,009	5,762	832	18:0 (283)	18:1 (281)	LPC 18:0 (508)	PC 18:0/18:1	—	—	—
					20:1 (309)	16:0 (255)	LPC 20:1 (534)	PC 20:1/16:0	—	—	—
PC 36:4	4,751*^f^*	4,246	3,212	826	16:0 (255)	20:4 (303)	LPC 16:0 (480)	PC 16:0/20:4	—	—	—
					18:2 (279)	18:2 (279)	LPC 18:2 (504)	PC 18:2/18:2	—	—	—
PC 38:4	5,507*^f^*	4,998	3,812	854	18:0 (283)	20:4 (303)	LPC 18:0 (508)	PC 18:0/20:4	—	—	—
PC 40:1	28*^f^*	42	18	888	16:0 (255)	**24:1** (365)	LPC 16:0 (480)	PC 16:0/24:1	—	—	—
PC 42:1	36*^f^*	58	12	916	**26:1** (393)	16:0 (255)	LPC 26:1 (618)	PC 26:1/16:0	—	—	—
					**24:0** (367)	18:1 (281)	LPC 24:0 (592)	PC 24:0/18:1	—	—	—
PC 42:2	29*^f^*	45	14	914	**24:0** (367)	18:2 (279)	LPC 24:0 (592)	PC 24:0/18:2	—	—	—
					**24:1** (365)	18:1 (281)	LPC 24:1 (590)	PC 24:1/18:1	—	—	—
PC 42:3	21*^f^*	29	13	912	**24:2** (363)	18:1 (281)	LPC 24:2 (588)	PC 24:2/18:1	—	—	—
PC 44:1	30*^f^*	45	1	944	**26:0** (395)	18:1 (281)	LPC 26:0 (620)	PC 26:0/18:1	—	—	—
PC 44:2	23*^f^*	38	3	942	**26:1** (393)	18:1 (281)	LPC 26:1 (618)	PC 26:1/18:1	—	—	—
PE 34:4	83	106	67	710	14:0 (227)	20:4 (303)	LPE 14:1 (424)	PE 14:0/20:4	—	—	—
SM 32:1	1,504	1,563*^h^*	1,135	719	—		—	—	16:0 (255)	d16:1 (421)	SM d16:1/16:0
					—		—	—	14:0 (255)	d18:1 (449)	SM d18:1/14:0
SM 34:1	20,208*^h^*	13,219	11,769	747	—		—	—	16:0 (255)	d18:1 (449)	SM d18:1/16:0
SM 34:2	2,295	3,139*^f^*	2,099	745	—		—	—	16:0 (255)	d18:2 (447)	SM d18:2/16:0
SM 44:1	248*^f^*	193*^h^*	42	887	**—**		—	—	**26:0** (395)	d18:1 (449)	SM d18:1/26:0
SM 44:2	421	356*^f^*	154	885	**—**		—	—	**26:1** (393)	d18:1 (449)	SM d18:1/26:1

a Each PL species observed by semi-quantitative analysis is represented by the total carbon and double bond number of two fatty acyl moieties (PC) or by the long-chain base and an *N-*acyl moiety (SM).

b PC 38:0 and SM 44:3 could not be assigned because of the absence of a spectrum corresponding to 1-acyl LPC and *N*-acyl moiety in LC-MS^3^ analysis, respectively.

c The quantity of each PL species in the CCALD, AMN, and control fibroblasts picomoles per milligram of protein).

d The *m/z* of the first precursor ion was used to analyze the structure of each PL species in MS^3^ using the negative ion mode.

e Notation in bold indicates fatty acyl moieties with no less than 24 carbons. FA, LPC, and SPC represent fatty acyl-moiety, LPC, and sphingosylphosphorylcholine (SPC), respectively.

f *P* < 0.05 versus control (Dunnett T3 post hoc test).

g *P* < 0.05 versus AMN (Dunnett T3 post hoc test).

h *P* < 0.01 versus control (Dunnett T3 post hoc test).

### Quantitative analysis of acyl-CoA species in ABCD1-deficient HeLa cells

**Fig. 3.**
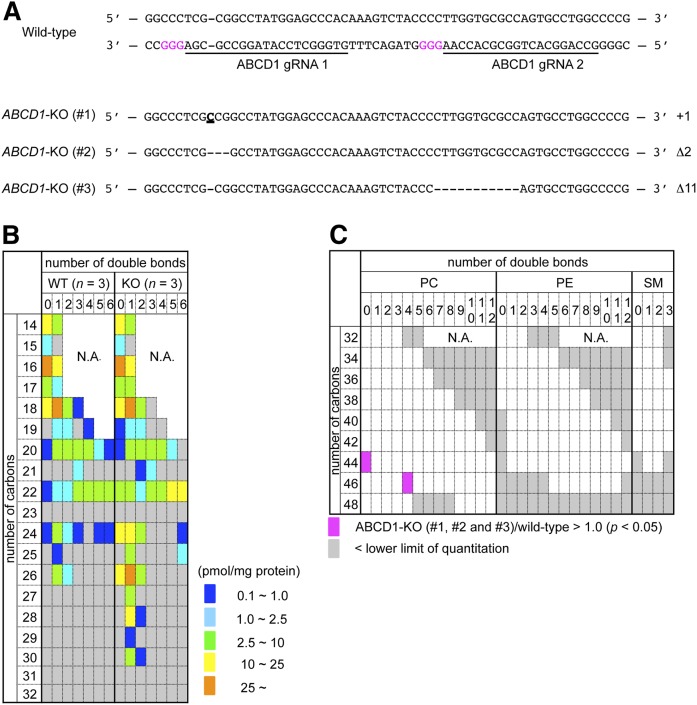
Quantity of each acyl-CoA species in the ABCD1-KO HeLa cells. A: Generation of ABCD1-KO HeLa cells with the CRISPR/Cas9 system. Partial genomic sequences for *ABCD1* are shown. The 20 bp target sequences of the gRNA are underlined. The protospacer adjacent motif (PAM) is represented in magenta. The bold and underlined nucleotide represents an insertion (#1), while the dashes (#2 and #3) represent deletions. These mutations all result in a frameshift and ABCD1 KO. B: The quantity of each acyl-CoA species in the ABCD1-KO HeLa cell lines (#1 to #3) compared with three independent clones of WT HeLa cells. The ratio of peak area for each acyl-CoA/D_31_-16:0-CoA was used to calculate the amount of each acyl-CoA species and the mean quantity of each acyl-CoA species is represented with a color key. The data are also summarized in Table 4. Acyl-CoA species observed to be present below the quantitation range are indicated in gray. C: Each PC, PE, and SM species present in significantly higher quantities (magenta) in the ABCD1-KO HeLa cell lines (#1 to #3) compared with three independent clones of WT HeLa cells were classified according to the number of carbons and double bonds in the two acyl moieties. No PL species were present in significantly lower quantities than in the control. PL species observed to be present below the quantitation range are indicated in gray. The species for which there were no MRM channels designed are indicated as N.A. The quantity of each PL species in the ABCD1-KO HeLa cells is listed in supplemental Table S2

Skin fibroblast cells from X-ALD patients senesce and show limited cell proliferation. To further examine the effect of ABCD1 deficiency on cellular acyl-CoA metabolism in other cell types, we generated three independent ABCD1-KO HeLa cell lines with the CRISPR/Cas9 system (**Fig. 3A**) (21) and compared them with three independent clones of WT HeLa cells. Penetrance differences were observed in the quantity of each acyl-CoA species, although mono- and di-unsaturated VLCFA-CoA species were significantly accumulated in all three ABCD1-KO HeLa cells (Fig. 3B, **Table 4**), as was likely observed in the X-ALD fibroblasts (Fig. 2A, Table 2). The amount of 26:1-CoA was highest among the VLCFA-CoA species in ABCD1-KO (#1 to #3) and control HeLa cells (Fig. 3B, Table 4). Through quantitative and structural PL analysis, only two PC species (PC 44:0 and 46:4), which contain 26:0- and 28:0-fatty acyl moieties, respectively, were found to be present in significantly higher quantities in the ABCD1-KO HeLa cells (#1 to #3) (Fig. 3C, supplemental Fig. S2, **Table 5**, supplemental Table S2). Taken together, these results show that the metabolism of mono- and di-unsaturated VLCFA-CoAs is mainly affected through ABCD1 dysfunction in both fibroblasts and HeLa cells, and that the accumulated VLCFA-CoAs are preferably incorporated into PC and SM species.

**TABLE 4.** The quantity of each acyl-CoA species in the ABCD1-KO HeLa cells

	WT (n = 3)		KO #1		KO #2		KO #3	
Signals	Amount*^a^*	Percent	Amount*^a^*	Percent	Amount*^a^*	Percent	Amount*^a^*	Percent
14:0-CoA	15.3 ± 2.3	5.0	13.3	2.7	9.7	3.1	16.4	2.7
14:1-CoA	3.3 ± 1.1	1.1	4.0	0.8	2.6	0.8	6.2	1.0
15:0-CoA	1.7 ± 1.5	0.6	1.6	0.3	3.1	1.0	4.4	0.7
16:0-CoA	73.3 ± 17.7	24.2	88.4	17.8	74.2	24.0	103.1	17.2
16:1-CoA	22.2 ± 3.9	7.3	18.8	3.8	22.8	7.4	24.6	4.1
17:0-CoA	4.3 ± 0.3	1.4	5.8	1.2	5.1	1.7	8.9	1.5
17:1-CoA	2.4 ± 0.9	0.8	2.0	0.4	3.0	1.0	4.9	0.8
18:0-CoA	13.9 ± 1.8	4.6	26.0	5.2	10.2	3.3	21.6	3.6
18:1-CoA	86.6 ± 18	28.5	77.5	15.6	70.7	22.8	106.6	17.8
18:2-CoA	7.6 ± 1.4	2.5	5.7	1.1	5.1	1.6	9.6	1.6
19:1-CoA	1.6 ± 0.2	0.5	2.3	0.5	1.6	0.5	2.7	0.4
19:2-CoA	1.6 ± 0.7	0.5	1.9	0.4	2.0	0.6	3.0	0.5
20:0-CoA	0.5 ± 0.5	0.2	0.4	0.1	0.0	0.0	2.5	0.4
20:1-CoA	6.5 ± 1.0	2.1	7.3	1.5	7.2	2.3	8.6	1.4
20:2-CoA	5.6 ± 0.8	1.8	6.7	1.3	5.1	1.7	5.9	1.0
20:3-CoA	7.0 ± 2.0	2.3	11.7	2.4	5.6	1.8	8.9	1.5
20:4-CoA	7.9 ± 3.0	2.6	11.5	2.3	5.5	1.8	12.3	2.0
20:5-CoA	1.9 ± 0.8	0.6	1.8	0.4	1.0	0.3	4.4	0.7
21:3-CoA	1.2 ± 0.7	0.4	2.2	0.4	1.5	0.5	2.6	0.4
22:0-CoA	0.3 ± 0.5	0.1	1.9	0.4	1.6	0.5	4.3	0.7
22:1-CoA	2.4 ± 0.5	0.8	3.6	0.7	3.2	1.0	6.8	1.1
22:2-CoA	1.6 ± 0.3	0.5	1.9	0.4	1.7	0.6	1.1	0.2
22:3-CoA	3.3 ± 1.2	1.1	5.4	1.1	3.6	1.2	2.5	0.4
22:4-CoA	2.8 ± 0.6	0.9	4.6	0.9	2.4	0.8	6.7	1.1
22:5-CoA	8.3 ± 2.7	2.7	13.6	2.7	6.0	1.9	19.1	3.2
22:6-CoA	7.7 ± 2.9	2.5	10.2	2.1	4.3	1.4	19.7	3.3
24:0-CoA	0.6 ± 0.5	0.2	7.6	1.5	6.1	2.0	19.6	3.3
24:1-CoA*	3.1 ± 1.1	1.0	13.1	2.6	10.8	3.5	25.2	4.2
24:2-CoA	1.2 ± 0.1	0.4	1.9	0.4	1.8	0.6	3.0	0.5
25:1-CoA*	0.4 ± 0.5	0.1	4.2	0.8	1.6	0.5	5.0	0.8
25:6-CoA	n.q.	0.0	1.2	0.2	n.q.	0.0	2.5	0.4
26:0-CoA	n.q.	0.0	19.5	3.9	1.6	0.5	21.7	3.6
26:1-CoA*	4.3 ± 0.4	1.4	74.8	15.1	22.7	7.3	71.6	11.9
26:2-CoA*	1.2 ± 0.2	0.4	8.4	1.7	3.6	1.2	7.6	1.3
27:1-CoA	n.q.	0.0	5.5	1.1	n.q.	0.0	3.1	0.5
28:1-CoA	n.q.	0.0	23.1	4.7	2.4	0.8	16.5	2.7
28:2-CoA	n.q.	0.0	1.5	0.3	n.q.	0.0	n.q.	0.0
30:1-CoA	n.q.	0.0	4.0	0.8	n.q.	0.0	5.3	0.9
Others	1.8 ± 0.7	0.6	13.1	2.6	10.8	3.5	25.2	4.2
Total	303.5 ± 20.1	100.0	496.1	100	309.6	100	599.8	100
VLCFA-CoA (DB*^b^* = 0)	0.6 ± 0.5	0.2	27.1	5.5	7.8	2.5	41.3	6.9
VLCFA-CoA (DB = 1)*^c^*	7.8 ± 1.8	2.6	124.6	25.1	37.5	12.1	126.7	21.1
VLCFA-CoA (DB = 2)*^c^*	2.4 ± 0.4	0.8	11.9	2.4	5.4	1.7	10.6	1.8
VLCFA-CoA (DB = 6)	n.q.	0.0	1.2	0.2	n.q.	0.0	2.5	0.4
Total of VLCFA-CoA*^c^*	10.8 ± 2.1		164.8		50.6		181.1	

a Data are expressed as picomoles per milligram of protein. The ratio of peak area for each acyl-CoA/D_31_-16:0-CoA was used to calculate the amount of each acyl-CoA species. n.q., below the quantitation range.

b DB represents the number of double bonds in the fatty acyl moieties.

c *P* < 0.05, ABCD1-KO (#1, #2, and #3) vs WT (n = 3) (*t*-test).

**TABLE 5.** Structure of the PL species present in quantities significantly higher in the ABCD1-KO HeLa cells

	Amount*^b^*						
Signals*^a^*	WT (n = 3)	KO (n = 3)	(*m/z*)*^c^*	*sn*-1 FA (*m/z*)*^d^*	*sn*-2 FA (*m/z*)*^d^*	1-Acyl LPC (*m/z*)*^d^*	Molecular Species
PC 44:0	n.q.	3	946	**28:0** (423)	16:0 (255)	LPC 28:0 (648)	PC 28:0/16:0
				**26:0** (395)	18:0 (283)	LPC 26:0 (620)	PC 26:0/18:0
PC 46:4	n.q.	2	966	**26:0** (395)	20:4 (303)	LPC 26:0 (620)	PC 26:0/20:4

a Each PL species observed in semi-quantitative analysis is represented by the total carbon and double bond number of two fatty acyl moieties (PC).

b The quantity of each PL species in the three WT and three ABCD1-KO (KO) HeLa cell lines (picomoles per milligram of protein). n.q., below the quantitation range.

c The *m/z* of the first precursor ion was used to analyze the structure of each PL species in MS^3^ using the negative ion mode.

d Notation in bold indicates fatty acyl moieties with no less than 24 carbons.

### Hexacosenoyl-CoA (26:1-CoA) is not efficiently synthesized from 26:0-CoA in ABCD1-deficient HeLa cells

**Fig. 4.**
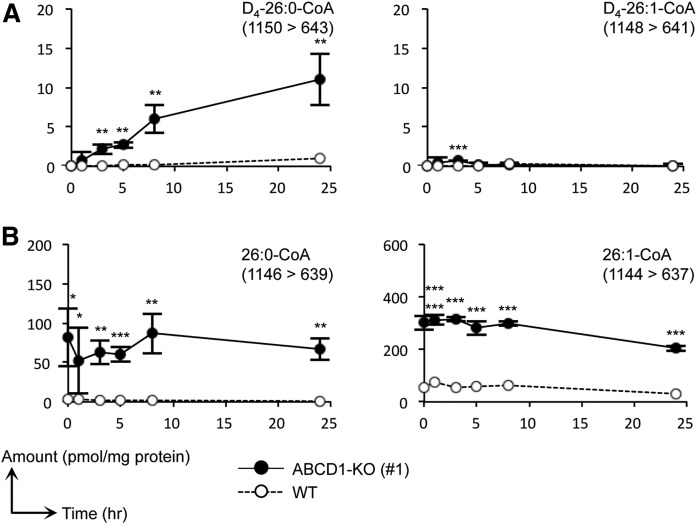
Metabolic analysis of VLCFA-CoA species using FA D_4_-26:0. Metabolic profiles of 26:0- and 26:1-CoA species containing a deuterium-labeled (A) or nonlabeled (B) acyl moiety in ABCD1-KO (#1) and WT HeLa cells (WT). Cells were cultured in medium containing 10% FBS and were treated with 30 μM of FA D_4_-26:0 and the MβCD complex. Cells were harvested at 1, 3, 5, 8, and 24 h after treatment. We confirmed that the retention times of each deuterium-labeled acyl-CoA species were almost identical with those of the corresponding nonlabeled VLCFA-CoA species. The MRM transitions (Q1/Q3) used are indicated in each panel. Data represent the mean ± SD, n = 3. Statistical analyses were performed with the Student’s *t*-test; *P < 0.05, **P < 0.01, ***P < 0.001 for ABCD1-KO versus WT HeLa cells

To elucidate the route of 26:1-CoA synthesis, we first examined the possibility of 26:0-CoA being a precursor of 26:1-CoA. To this end, we labeled the ABCD1-KO HeLa cells with deuterium-labeled hexacosanoic acid (FA D_4_-26:0) and analyzed the endogenous rate of synthesis of D_4_-26:0- and D_4_-26:1-CoA. The quantity of D_4_-26:0-CoA in the ABCD1-KO cells increased in a time-dependent manner in the 24 h following the exogenous administration of 30 μM of FA D_4_-26:0, while the levels of D_4_-26:1-CoA were not significantly altered by this treatment (**Fig. 4A**). In contrast, D_4_-26:1-CoA was present in much smaller quantities in the ABCD1-KO cells compared with the unlabeled 26:1-CoA, and it was not accumulated in a time-dependent manner (Fig. 4A, B). These results suggest the presence of acyl-CoA synthases, which utilize FA 26:0 as a substrate, although 26:1-CoA was not efficiently synthesized from 26:0-CoA through a FA desaturation process.

### Hexacosenoyl-CoA (26:1-CoA) is efficiently synthesized from 18:1-CoA in ABCD1-deficient HeLa cells

**Fig. 5.**
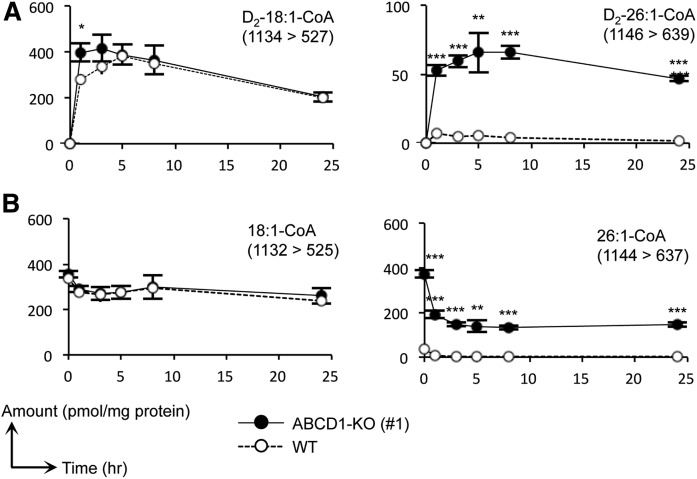
Metabolic analysis of VLCFA-CoA species using FA D_2_-18:1. Metabolic profiles of each deuterium-labeled 18:1- and 26:1-CoA species (A) and nonlabeled 18:1- and 26:1-CoA species (B) were examined in ABCD1-KO (#1) and WT HeLa cells. The metabolic profiles of the other labeled and nonlabeled acyl-CoA species are depicted in supplemental Fig. S3. Cells were cultured in medium containing 10% FBS and treated with 30 μM of FA D_2_-18:1 and the MβCD complex. The cells were harvested at 1, 3, 5, 8, and 24 h after treatment. The MRM transitions (Q1/Q3) used are indicated in each panel. Statistical analyses were performed with the Student’s *t*-test; *P < 0.005, **P < 0.01, ***P < 0.001 for ABCD1-KO versus WT HeLa cells

It was previously shown that FA 26:0 is intracellularly synthesized from FA 18:0 through FA elongation, and the protein, elongation of very long-chain FAs 1 (ELOVL1), plays a critical role in the synthesis of VLCFAs (1, 26). Thus, we next examined whether 26:1-CoA can be synthesized through the FA elongation machinery from the 18:1-CoA that is abundantly present as a long-chain fatty acyl-CoA species in cells (Figs. 2A, 3B; Tables 2, 4). To this end, we labeled the ABCD1-KO HeLa cells with deuterium-labeled oleic acid (FA D_2_-18:1) and analyzed the endogenous rate of synthesis of D_2_-24:1- and D_2_-26:1-CoA as well as D_2_-18:1-CoA (**Fig. 5**). The D_2_-18:1-, D_2_-24:1-, and D_2_-26:1-CoA levels immediately increased and reached a plateau within an hour after treatment with FA D_2_-18:1 in ABCD1-KO HeLa cells (Fig. 5A), showing that 26:1-CoA can be efficiently synthesized from 18:1-CoA through the endogenous FA elongation process after the activation of FA 18:1 by acyl-CoA synthetase. Note that the levels of D_2_-18:1-CoA and D_2_-26:1-CoA did not significantly change, while D_2_-24:1-CoA markedly decreased in the ABCD1-KO cells during the first 1–8 h after treatment (supplemental Fig. S3A). These results are consistent with the results obtained for the PL species with VLCFAs in their acyl moieties in ABCD1-KO HeLa cells (Table 5). Thus, C_24_ fatty acyl-CoAs are transferred to PC species more efficiently than C_26_ fatty acyl-CoAs. Interestingly, the quantity of nonlabeled 18:1-CoA did not significantly alter during the observation period following treatment (Fig. 5B); in contrast, the nonlabeled VLCFA-CoAs (24:1-, 24:2-, 26:1-, 26:2-, 28:1-, and 28:2-CoA) were significantly reduced within the first hour after treatment and remained at low levels (∼150 pmol/mg protein) after treatment with 30 μM of FA D_2_-18:1 (Fig. 5B, supplemental Fig. S3B). These results suggest that FA 18:1 itself or the metabolites of FA 18:1 could negatively regulate the endogenous synthesis of VLCFA-CoAs. Given that the pool of each fatty acyl-CoA species was maintained by some homeostatic machinery, it may be also possible that the amount of D_2_-26:1-CoA increased and then nonlabeled 26:1-CoA decreased inversely.

### The pool of monounsaturated VLCFA-CoAs is turned over as efficiently as 18:1-CoA in both WT and ABCD1-deficient HeLa cells

**Fig. 6.**
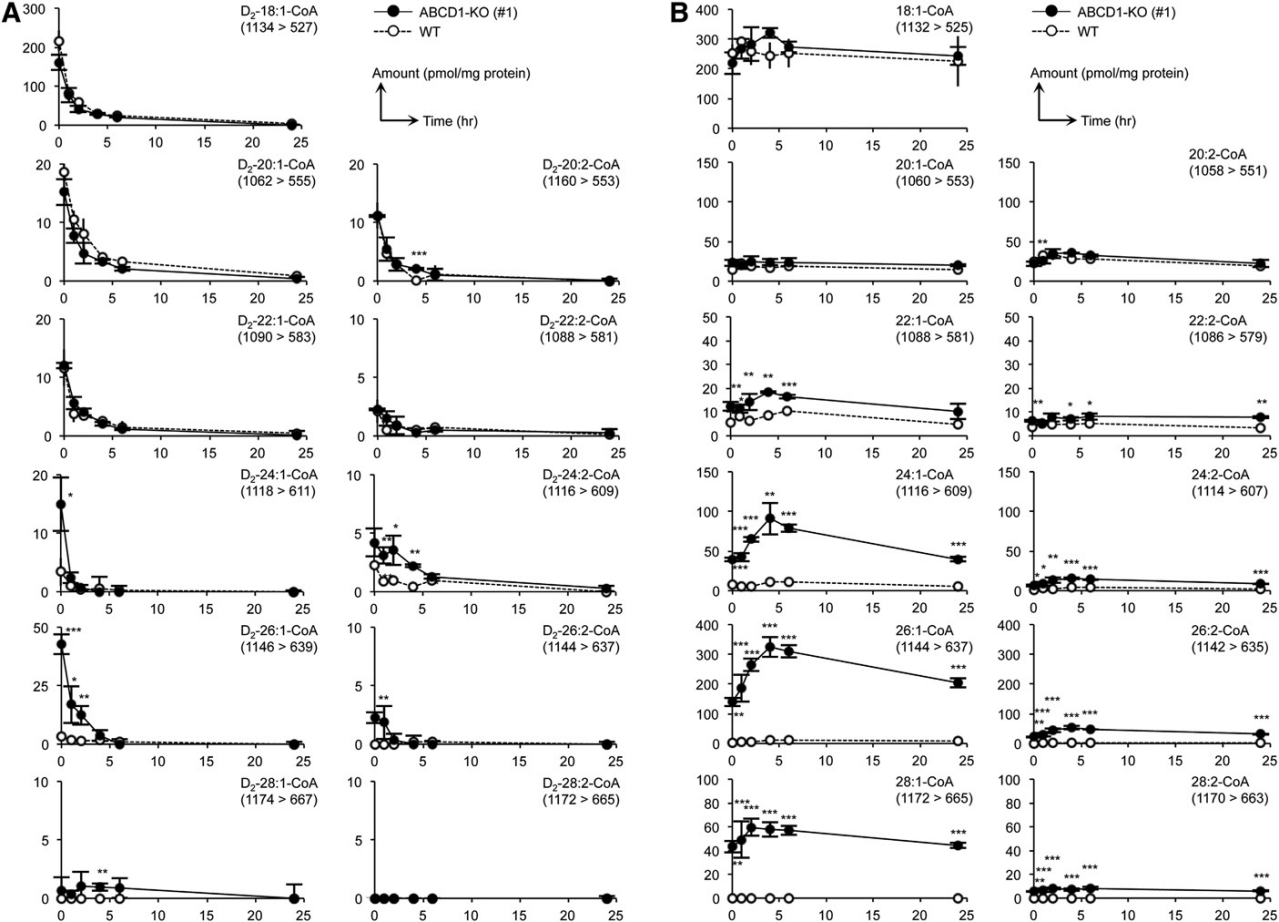
Pulse-chase analysis of VLCFA-CoA species using FA D_2_-18:1. Metabolic profiles of 15 acyl-CoA species containing a deuterium-labeled (A) or nonlabeled (B) acyl moiety in ABCD1-KO (#1) and WT HeLa cells were examined. Cells were cultured in medium containing 10% FBS and treated with 30 μM of FA D_2_-18:1 and the MβCD complex for an hour, after which the medium was replaced with fresh medium containing 10% FBS. The cells were then harvested at 1, 2, 4, 6, and 24 h after treatment. Acyl-CoA species were extracted and analyzed by LC-MS/MS analysis. The MRM transitions (Q1/Q3) used are indicated in each panel. Statistical analyses were performed with Student’s *t*-test; *P < 0.05, **P < 0.001, ***P < 0.001 for ABCD1-KO versus WT HeLa cells

It is believed that acyl-CoA species are stored intracellularly and function as essential intermediates for FA metabolism. However, little is known regarding the metabolic turnover of each acyl-CoA species. This is at least partially due to the lack of a highly sensitive and precise quantitative method for acyl-CoA detection. To clarify the difference in metabolic turnover between each acyl-CoA species under the ABCD1-deficient condition, a pulse-chase study was conducted using deuterium-labeled oleic acid (FA D_2_-18:1) in WT and ABCD1-KO HeLa cells (#1). In ABCD1-KO HeLa cells, 10 acyl-CoA species with deuterium-labeled acyl moieties (D_2_-18:1-, D_2_-20:1-, D_2_-20:2-, D_2_-22:1-, D_2_-22:2-, D_2_-24:1-, D_2_-24:2-, D_2_-26:1-, D_2_-26:2-, and D_2_-28:1-CoA) were observed within 1 h after labeling (**Fig. 6A**). Among these 10 deuterium-labeled acyl-CoA species, the D_2_-24:1-, D_2_-24:2-, D_2_-26:1-, and D_2_-26:2-CoA species were significantly accumulated compared with the WT HeLa cells, confirming the critical roles of ABCD1 in VLCFA-CoA metabolism (Fig. 6A). Most of the deuterium-labeled acyl-CoA species decreased immediately after medium replacement (Fig. 6A). In contrast, D_2_-28:1-CoA levels did not significantly change within at least the first 6 h after medium replacement and decreased gradually over the following 18 h (Fig. 6A). This suggests that the elongation process from 18:1-CoA to 26:1-CoA is highly efficient, while the conversion from 26:1-CoA to 28:1-CoA is not. To further analyze the VLCFA-CoA metabolism quantitatively, we conducted regression analysis by applying the exponential model, thereby obtaining data on the half-life of each acyl-CoA species. The half-life of most deuterium-labeled acyl-CoA species ranged from 0.5 to 2 h, and the differences in this value between the WT and ABCD1-KO HeLa cells were no more than 10% (18:1-CoA, 20:2-CoA, 22:1-CoA, 24:1-CoA, and 26:1-CoA; **Table 6**). Notably, the half-life of 26:1-CoA was almost identical with that of 18:1-CoA in both the WT and ABCD1-deficient HeLa cells, suggesting that these VLCFA-CoAs are metabolized with high efficiency even in the absence of ABCD1. Interestingly, the half-life of 24:1-CoA was almost half that of the other acyl-CoA species (Table 6), supporting the hypothesis that C_24_ fatty acyl-CoAs are transferred to PC species more efficiently than to C_26_ fatty acyl-CoAs in ABCD1-KO HeLa cells, resulting in the accumulation of PC species with C_24_ fatty acyl moieties as described in the previous section. Taken together, these results suggest that VLCFA-CoA species with one or two double bonds are efficiently metabolized, possibly through being incorporated into PC and SM. It is notable that while the quantities of nonlabeled VLCFA-CoAs (22:1-CoA, 24:1-CoA, 24:2-CoA, 26:1-CoA, 26:2-CoA, 28:1-CoA, and 28:2-CoA) initially increased during the first 4 h and then gradually decreased over the remainder of the chase period, those of nonlabeled 18:1-CoA and 20:1-CoA were not significantly altered (Fig. 6B). These results may be attributable to certain unknown and unstable factors in FBS, which stimulate the synthesis and the metabolic flux of VLCFAs.

**TABLE 6.** Regression analysis of FA metabolism in the ABCD1-KO HeLa cells

Acyl-CoA Species	Genotype	Coefficient of Determination (*r*^2^^2^)	[A_0_]*^a^*	−*^a^*	*t*_1/2_ (h)*^b^*
D_2_-18:1-CoA	WT	0.895	199.7	−0.661	1.05
	KO #1	0.962	155.9	−0.626	1.11
D_2_-20:1-CoA	WT	0.751	17.8	−0.390	1.78
	KO #1	0.811	13.9	−0.575	1.21
D_2_-20:2-CoA	WT	0.913	10.7	−0.656	1.06
	KO #1	0.867	10.7	−0.688	1.01
D_2_-22:1-CoA	WT	0.601	9.9	−0.586	1.18
	KO #1	0.940	11.7	−0.541	1.28
D_2_-22:2-CoA	WT	0.514	1.8	−0.623	1.11
	KO #1	0.634	2.4	−0.311	2.23
D_2_-24:1-CoA	WT	0.914	3.5	−1.311	0.53
	KO #1	0.952	14.8	−1.452	0.48
D_2_-24:2-CoA	WT	0.531	2.0	−0.400	1.73
	KO #1*^c^*	—	—	—	—
D_2_-26:1-CoA	WT	0.902	2.9	−0.651	1.06
	KO #1	0.869	42.5	−0.653	1.06
D_2_-26:2-CoA	WT*^d^*	—	—	—	—
	KO #1	0.596	2.7	−0.898	0.77
D_2_-28:1-CoA	WT*^c^*	—	—	—	—
	KO #1*^c^*	—	—	—	—

a The exponential model {[A] = [A_0_]exp(−*kt*)} was applied to the quantity of each deuterium-labeled acyl-CoA species during the 0–4 h chase period to estimate the metabolic state of each acyl-CoA species. [A], concentration of each acyl-CoA species; [A_0_], initial concentration of each acyl-CoA species; *k*, reaction rate constant; *t*, time (hours).

b The half-life (*t*_1/2_) was based on *t*_1/2_ = ln (2)/*k*.

c The exponential model could not be applied because the quantity of deuterium-labeled CoA did not decrease monotonically during the initial 2 h (D_2_-24:2-CoA) or 4 h (D_2_-28:1-CoA).

d The exponential model could not be adequately applied because of the low coefficient of determination (*r*^2^ < 0.5).

## DISCUSSION

In this study, it was clarified that 26:1-CoA was the most abundant VLCFA-CoA species present in both of the X-ALD fibroblasts and in the ABCD1-KO HeLa cells (Figs. 2A, 3B; Tables 2, 4). Most of the 26:1-CoA was shown to be synthesized from 18:1-CoA via the FA elongation pathway instead of synthesis from 26:0-CoA via the desaturation pathway (Figs. 4, 5). Thus, the quantity of FA 18:1 present as the initial substrate will affect the VLCFA-CoA profile in ABCD1-deficient cells. Furthermore, the activity of acyl-CoA synthase, which synthesizes 18:1-CoA from FA 18:1, will also affect the quantity of VLCFA-CoAs in ABCD1-deficient cells.

It is clinically important to develop biochemical markers to estimate the progression of X-ALD. In the present study, we observed significant differences in the amount of polyunsaturated fatty acyl-CoAs (20:2-, 22:5-, and 24:5-CoA) and two PC species with saturated fatty acyl-moieties (PC 32:0 and 38:0) between CCALD and AMN fibroblasts (Fig. 2A, C; Tables 2, 3; supplemental Table S1). Considering that α-linolenic acid (FA 18:3) is converted to FA 22:5 and FA 24:5 resulting in docosahexaenoic acid (FA 22:6) (2), these results may indicate the difference in the efficiency of the metabolism of polyunsaturated FAs involving several elongases and desaturases between CCALD and AMN fibroblasts. In this study, we analyzed human fibroblasts from skin biopsy samples of two sibling X-ALD patients with an identical mutation on the *ABCD1* gene (c1825G>A); one X-ALD fibroblast was from a patient with CCALD phenotype, and the other X-ALD fibroblast was from a patient with AMN phenotype. We could not find significant differences in the amount of each acyl-CoA and PL species between these two X-ALD fibroblasts (data not shown). Further analysis including the dynamics of VLCFA metabolism is necessary to discover the novel features underlying differences in the severity of clinical symptoms between X-ALD subtypes.

Several experiments using stable isotope labeling have been conducted to analyze the fate of acyl-CoA species. For example, U-^13^C-palmitic acid was administered to rabbits and ^13^C isotope enrichment based on the ratio of 16:0-CoA to U-^13^C-16:0-CoA was determined in the muscle tissues by LC-MS (27). Similarly, deuterium-labeled 4-hydroxynonenal was used to analyze the catabolism of 4-hydroxy acids (C_4_ to C_11_) (28). However, the intracellular kinetics of each long-chain fatty acyl-CoA species has not yet been analyzed. In this study, we precisely analyzed the kinetics of each acyl-CoA species with pulse-chase experiments using FA D_2_-18:1. Interestingly, the kinetics of 18:1-CoA and 26:1-CoA were almost identical, and 24:1-CoA was turned over twice as rapidly as 18:1-CoA in both the WT and ABCD1-deficient cells (Table 6). Possible explanations for this finding could be that VLCFA-CoAs are rapidly released from the cells or that VLCFA-CoAs are efficiently introduced into PC and SM species in the absence of ABCD1. To address this issue, we conducted the pulse-chase experiments using D_2_-FA 18:1 and harvested cells at 0, 1, 2, 4, 6, and 24 h after the replacement of medium, and found that the amount of D_2_-PC 42:2, D_2_-PC 44:2, and D_2_-SM 42:2 were increased in a time-dependent manner. Especially, the amount of D_2_-SM 42:2 was much higher than that of D_2_-PC 42:2 and D_2_-PC 44:2 (data not shown). These results indicate that VLCFA-CoA is preferably introduced into sphinganine to form ceramide and SM in HeLa cells. We observed significant accumulation of VLCFA-containing PC and SM in both fibroblasts and ABCD1-deficient HeLa cells. VLCFA moieties, such as 24:1- or 26:1-fatty acyl, are located at the *sn*-1 position of the glycerol backbone of PC or bind to the amine of the long-chain sphingoid base of SM. Therefore, these results strongly suggest that 2-acyl lysophosphatidylcholine (LPC) acyltransferases and ceramide synthase are likely involved in the synthesis of VLCFA-containing PC and SM, respectively (29, 30).

Total FAs have been conventionally quantified so far by analyzing the methyl-esterified acids from both free FA and esterified FAs (9). Upon comparison of these acyl-CoA profiles in the X-ALD fibroblasts with the FA profiles previously reported (10, 31), it was clear that the relative abundance of each acyl-CoA was significantly different to that of the corresponding FA. Among the VLCFA-CoAs, for example, 26:1-CoA was the most abundantly concentrated in X-ALD fibroblasts and ABCD1-KO HeLa cells and the level of 26:1-CoA was 5- to 6-fold higher than that of 26:0-CoA, while the amount of total FA 26:1 was similar to that of FA 26:0 (10, 31). As another example, the quantities of 26:0-CoA and 24:0-CoA were almost identical, although the amount of total FA 26:0 was much lower than that of FA 24:0 in the X-ALD fibroblasts (10, 31). These discrepancies may be explained by the endogenous forms of the VLCFAs present; 26:0-CoA may be more efficiently transferred into complex lipids such as PL and then stored compared with 26:1-CoA. It is likely that 24:0-CoA may be a preferred substrate for PL synthesis compared with 26:0-CoA.

In previous studies, odd-numbered long-chain fatty acyl-CoA species, such as 17:0-CoA, were used as the IS (22, 23). However, significant levels of PLs with odd-numbered FAs are observed in mammalian tissues as we have reported previously (8, 32), raising the possibility that the total amount of IS in each sample is influenced by the endogenous odd-numbered fatty acyl-CoA species. To circumvent this issue, we chemically synthesized D_31_-16:0-CoA as the IS compound. As expected, significant amounts of odd-numbered acyl-CoA species were observed in both the fibroblasts and HeLa cells. For instance, the proportion of 17:0-CoA to total acyl-CoA quantified was 2.6–2.9% (11.4–42.1 pmol/mg protein) in the fibroblasts and 1.1–1.4% (1.8–2.5 pmol/mg protein) in the ABCD1-KO HeLa cells, showing the advantages of stable isotopically labeled fatty acyl-CoA in the quantitative analysis of acyl-CoA species by LC-MS. In this study, D_31_-16:0-CoA was used as the IS compound and the peak area of each acyl-CoA species was normalized by the peak area of D_31_-16:0-CoA. Because each acyl-CoA did not co-elute with the IS, our present method corresponds to the level 3 type of quantitation as defined by the Lipidomics Standards Initiative (https://lipidomics-standards-initiative.org). To estimate the difference of ionization efficiency between each long chain acyl-CoA and VLCFA-CoA species, we generated the two dilution series of 17:0-CoA and 26:0-CoA and analyzed the peak area of each analyte. We found that the electrospray ionization efficiency of 26:0-CoA was almost one-ninth of 17:0-CoA (data not shown). Because both acyl chain length and retention time of D_31_-16:0-CoA are close to those of 17:0-CoA rather than 26:0-CoA, these results indicate that the amounts of VLCFA-CoA species with fewer number of double bonds in their acyl chain moiety were underestimated in this study, and the amount of 26:1-CoA may be much higher than that of 18:1-CoA in both X-ALD fibroblasts and ABCD1-deficient HeLa cells. The ionization efficiency can be influenced by ion suppression or ion enhancement depending on the mobile phase conditions and the presence of coeluting compounds (24), and it is desirable to prepare an IS compound for each acyl-CoA species for absolute quantitation. If stable isotopically labeled CoA is abundantly available, the preparation of each IS compound may be facilitated by a condensation reaction between each nonlabeled FA and the labeled CoA as conducted in this study.

In conclusion, we profiled the pool of intracellular acyl-CoA in X-ALD fibroblasts and ABCKD-KO HeLa cells and revealed that 26:1-CoA, but not 26:0-CoA, was the most abundantly accumulated VLCFA-CoA in both cell types. In addition, we conducted pulse-chase experiments using stable isotope-labeled FA and clarified that 26:1-CoA was efficiently synthesized from FA 18:1. Furthermore, we showed that 26:1-CoA is efficiently turned over in the absence of ABCD1, illustrating the efficiency of the ABCD1-independent machinery that metabolizes VLCFA-CoA.

## Supplementary Material

Supplemental Data
