# Supplementary material for: Hexacosenoyl-CoA is the most abundant very long-chain acyl-CoA in ATP binding cassette transporter D1-deficient cells
Source: J Lipid Res. 2020 Feb 19;61(4):523–36. doi: 10.1194/jlr.P119000325 (PMC7112142; doi:10.1194/jlr.P119000325)
Supplement: Supplemental Data [file supp_61_4_523__index.html]

Hexacosenoyl-CoA is the most abundant very long-chain acyl-CoA in ATP-binding cassette transporter D1-deficient cells — Profiling of acyl-CoA species in ABCD1-deficient cells — Hexacosenoyl-CoA is the most abundant very long-chain acyl-CoA in ATP binding cassette transporter D1-deficient cells — Supplemental Data 

# Hexacosenoyl-CoA is the most abundant very long-chain acyl-CoA in ATP binding cassette transporter D1-deficient cells

## Supplemental Data

- Supplemental Table S2 - Supplemental Table S2
- Supplemental Table S1 - Supplemental Table S1
- Supplemental Figure S3 - Supplemental Figure S3
- Supplemental Figure S2 - Supplemental Figure S2
- Supplemental Figure S1 - Supplemental Figure S1
