# Supplementary material for: Hexacosenoyl-CoA is the most abundant very long-chain acyl-CoA in ATP binding cassette transporter D1-deficient cells
Source: J Lipid Res. 2020 Feb 19;61(4):523–36. doi: 10.1194/jlr.P119000325 (PMC7112142; doi:10.1194/jlr.P119000325)
Supplement: Supplemental Data [file supp_P119000325_155112_2_supp_476153_q5pyyv.pdf]

Supplemental Table S2 The quantity of each PL species in the ABCD1-KO HeLa cells

Signals	wild-type (n = 3)[a]	KO #1[a]	KO #2[a]	KO #3[a]
pc32:0	2052.2 ± 100.1	1,874.0	2,078.4	3,112.8
pc32:1	3557.9 ± 122.0	3,272.8	3,530.3	4,200.0
pc32:2	2087.7 ± 572.3	2,084.6	1,371.6	1,035.7
pc34:0	1457.0 ± 272.1	936.1	1,116.7	1,371.3
pc34:1	3516.8 ± 138.4	3,271.8	4,205.6	5,160.9
pc34:2	4130.4 ± 542.3	4,000.3	3,784.0	3,746.6
pc34:3	807.8 ± 217.7	868.1	580.2	558.6
pc34:4	179.3 ± 44.3	165.3	148.2	177.9
pc34:5	12.3 ± 3.1	14.2	14.6	22.0
pc36:0	90.8 ± 44.9	34.5	32.9	43.9
pc36:1	2842.1 ± 116.9	2,583.0	2,762.7	3,208.7
pc36:2	3566.0 ± 93.9	3,351.6	3,763.4	4,277.3
pc36:3	3395.7 ± 523.0	3,508.9	2,613.8	2,507.5
pc36:4	2094.0 ± 209.2	1,843.0	1,832.8	1,882.1
pc36:5	1186.9 ± 152.9	976.9	1,006.0	1,500.8
pc36:6	94.8 ± 12.0	79.0	100.7	129.7
pc38:0	17.5 ± 0.6	20.6	10.3	14.0
pc38:1	318.5 ± 33.8	178.5	131.9	116.2
pc38:2	797.8 ± 117.7	734.7	614.5	315.3
pc38:3	1037.7 ± 262.9	1,370.0	457.7	520.3
pc38:4	1002.5 ± 176.0	791.3	762.0	1,426.4
pc38:5	2628.1 ± 56.8	1,967.8	2,386.2	2,920.4
pc38:6	1956.9 ± 122.2	1,483.5	1,685.6	1,888.1
pc38:7	282.4 ± 26.3	225.1	249.4	248.4
pc38:8	12.7 ± 3.8	6.8	11.8	9.7
pc40:0	3.7 ± 0.7	8.0	4.8	12.5
pc40:1	25.2 ± 1.0	42.0	24.0	29.3
pc40:2	107.1 ± 18.0	88.6	57.9	34.4
pc40:3	228.7 ± 88.8	170.0	120.2	36.6
pc40:4	407.1 ± 148.6	338.5	197.5	163.3
pc40:5	622.3 ± 71.7	493.6	449.7	620.0
pc40:6	711.9 ± 116.7	585.0	535.8	458.8
pc40:7	1018 ± 144.1	833.4	800.1	663.1
pc40:8	270.6 ± 79.0	184.3	274.9	282.6
pc40:9	33.1 ± 14.2	23.7	51.5	57.2
pc40:10	2.9 ± 1.1	2.7	3.0	4.2
pc42:0	1.1 ± 0.1	11.8	2.2	18.7
pc42:1	7.7 ± 0.3	37.1	11.0	30.6
pc42:2	23.2 ± 5.9	74.5	26.3	27.9
pc42:3	37.9 ± 14.6	47.8	19.1	12.1
pc42:4	48.4 ± 21.5	42.8	19.5	12.7
pc42:5	66.4 ± 12.8	45.0	31.0	45.9
pc42:6	98.8 ± 5.5	60.5	56.5	67.0
pc42:7	125.0 ± 12.1	82.8	75.2	63.3
pc42:8	91.6 ± 15.7	74.5	61.7	57.7
pc42:9	311.4 ± 79.5	196.9	295.5	301.1
pc42:10	271.8 ± 90.3	151.2	354.5	365.0
pc42:11	20.5 ± 8.1	14.3	33.6	37.5
pc44:0*	n.q.	3.4	0.1	4.8
pc44:1	1.1 ± 0.3	18.2	2.2	18.4
pc44:2	4.1 ± 1.3	36.6	6.3	17.1
pc44:3	6.8 ± 2.6	35.6	9.0	8.0
pc44:4	7.0 ± 2.6	12.7	3.5	3.4
pc44:5	7.3 ± 1.3	9.1	5.5	6.7
pc44:6	18.6 ± 4.7	15.7	10.9	12.5
pc44:7	26.9 ± 6.7	16.8	14.9	13.2
pc44:8	19.2 ± 4.5	13.8	12.1	9.7
pc44:9	16.8 ± 8.1	5.7	9.5	20.0
pc44:10	68.4 ± 15.6	40.9	57.0	49.5
pc44:11	115.4 ± 35.4	65.5	93.1	69.5
pc44:12	32.7 ± 11.6	18.3	38.4	27.7
pc46:1	n.q.	3.0	n.q.	2.7
pc46:2	0.1 ± 0.1	5.5	0.4	2.1
pc46:3	0.4 ± 0.3	4.9	0.6	1.6
pc46:4*	n.q.	1.1	0.2	5.0

Signals	wild-type ($n = 3$)[a]	KO #1[a]	KO #2[a]	KO #3[a]
pc46:5	2.0 ± 0.8	11.5	2.5	7.4
pc46:6	4.9 ± 1.6	10.9	3.9	5.1
pc46:7	2.2 ± 0.8	3.0	1.4	1.0
pc46:8	5.4 ± 2.2	4.4	3.2	2.5
pc46:9	3.3 ± 1.1	1.9	1.6	2.0
pc46:10	3.5 ± 1.2	1.2	1.6	3.8
pc46:11	3.6 ± 1.6	1.1	2.0	2.7
pc46:12	1.3 ± 0.3	0.5	0.8	0.8
pc48:5	0.0 ± 0.1	2.9	n.q.	2.7
pc48:6	0.5 ± 0.4	3.0	0.7	1.5
pc48:7	0.9 ± 0.4	1.6	0.6	n.q.
pc48:8	1.0 ± 0.3	3.3	0.8	0.6
pe32:0	329.1 ± 64.6	255.4	334.0	296.9
pe32:1	2018.3 ± 450.8	2,530.0	1,815.8	1,406.6
pe32:2	285.0 ± 154.7	392.8	196.8	119.6
pe34:0	317.1 ± 55.6	221.9	252.5	224.9
pe34:1	7827.0 ± 813.5	8,905.1	9,725.4	9,877.1
pe34:2	3652.3 ± 1354.4	4,936.8	3,262.1	2,648.2
pe34:3	313.7 ± 162.0	453.0	247.1	215.9
pe34:4	66.4 ± 27.2	106.1	86.0	75.1
pe34:5	2.9 ± 1.2	3.9	4.5	5.9
pe36:0	18.1 ± 4.6	5.7	4.3	6.9
pe36:1	7881.9 ± 243.4	7,515.3	8,571.9	9,239.2
pe36:2	9610.9 ± 1262.2	10,990.6	11,485.4	11,825.2
pe36:3	2743.3 ± 1150.7	3,791.8	2,565.6	3,082.9
pe36:4	2439.5 ± 840.2	3,564.9	2,821.8	3,489.3
pe36:5	729.6 ± 257.2	1,010.6	807.4	1,382.5
pe36:6	66.4 ± 22.5	103.0	110.9	117.0
pe36:7	2.4 ± 1.2	3.9	3.1	2.9
pe38:0	1.3 ± 0.8	0.5	0.8	0.8
pe38:1	318.0 ± 88.0	75.3	109.8	106.8
pe38:2	1244.1 ± 160.0	933.7	1,000.8	437.8
pe38:3	3159.9 ± 1033.0	4,289.3	1,880.9	1,643.5
pe38:4	7374.2 ± 1762.4	9,652.9	6,461.4	10,759.9
pe38:5	6857.4 ± 1601.2	8,597.9	7,584.1	12,269.4
pe38:6	2474.0 ± 735.6	3,224.2	3,338.1	4,003.6
pe38:7	388.1 ± 168.2	639.5	556.6	507.8
pe38:8	10.8 ± 6.3	15.0	13.9	5.1
pe40:1	57.8 ± 11.1	26.3	28.7	27.1
pe40:2	335.1 ± 38.3	101.4	109.1	41.5
pe40:3	610.2 ± 319.5	542.4	316.7	101.9
pe40:4	1209.1 ± 649.8	936.8	799.1	1,345.2
pe40:5	2490.7 ± 746.8	2,809.8	2,862.7	4,660.6
pe40:6	2063.7 ± 202.7	2,583.1	2,773.8	4,098.2
pe40:7	2213.3 ± 443.7	2,854.8	2,855.4	2,358.0
pe40:8	146.5 ± 51.9	231.1	169.3	137.4
pe40:9	12.4 ± 4.6	17.9	17.2	19.1
pe40:10	2.3 ± 1.6	3.7	2.7	2.2
pe42:1	18.9 ± 1.8	16.7	20.3	26.3
pe42:2	41.2 ± 4.9	29.9	28.5	19.0
pe42:3	67.4 ± 26.4	30.9	24.7	9.5
pe42:4	41.7 ± 26.2	28.6	19.3	18.5
pe42:5	104.2 ± 43.6	70.1	55.7	87.0
pe42:6	126.1 ± 38.1	89.3	102.5	121.1
pe42:7	156.6 ± 60.6	122.2	175.4	135.0
pe42:8	89.4 ± 39.2	104.7	82.4	58.9
pe42:9	84.6 ± 25.4	112.7	77.3	63.7
pe42:10	194.4 ± 93.0	220.4	232.7	146.1
pe42:11	9.2 ± 4.5	9.9	11.9	10.7
pe44:1	0.8 ± 0.1	4.4	1.2	6.5
pe44:2	2.5 ± 0.7	7.8	3.3	5.2
pe44:3	5.3 ± 1.6	7.7	3.8	3.6
pe44:4	2.2 ± 1.0	0.4	1.2	1.2
pe44:5	26.3 ± 9.8	19.5	9.3	8.2
pe44:6	18.2 ± 6.5	18.2	12.2	6.9
pe44:7	13.4 ± 1.3	6.9	9.4	3.9
pe44:8	11.4 ± 4.3	8.2	10.0	6.0
pe44:9	21.0 ± 9.4	13.1	14.0	10.0

Signals	wild-type (n = 3)[a]	KO #1[a]	KO #2[a]	KO #3[a]
pe44:10	19.1 ± 4.4	17.7	21.4	18.9
pe44:11	27.8 ± 7.7	31.1	36.4	23.4
pe44:12	21.9 ± 6.9	29.2	41.5	25.9
pe46:5	1.6 ± 0.6	4.2	1.9	4.3
pe46:6	4.4 ± 1.8	4.5	2.5	2.0
pe46:7	3.7 ± 0.8	3.2	2.4	1.7
pe46:8	3.1 ± 1.6	2.9	2.1	0.7
pe46:9	2.0 ± 1.2	1.1	0.8	0.0
pe46:10	1.4 ± 0.5	0.6	0.7	0.9
pe46:11	1.1 ± 0.6	0.2	0.9	0.8
sm32:0	163.9 ± 45.6	101.1	126.2	154.1
sm32:1	563.4 ± 72.1	677.6	703.9	635.3
sm32:2	13.5 ± 0.7	12.2	14.8	14.2
sm34:0	1206.9 ± 346.4	911.6	1,165.3	2,003.1
sm34:1	2736.3 ± 447.5	2,782.3	4,473.8	6,377.6
sm34:2	1154.7 ± 119.9	1,353.2	1,403.7	1,486.0
sm36:0	147.8 ± 68.1	67.0	80.4	83.1
sm36:1	487.2 ± 197.6	336.6	446.3	550.3
sm36:2	168.2 ± 51.7	119.3	144.8	164.9
sm36:3	10.7 ± 2.5	8.2	9.2	10.3
sm38:0	64.5 ± 43.9	28.6	43.6	38.3
sm38:1	196.0 ± 31.3	124.8	142.1	138.4
sm38:2	24.5 ± 2.9	23.7	26.0	23.6
sm38:3	6.9 ± 1.0	4.3	4.5	8.0
sm40:0	90.7 ± 53.4	7.4	10.4	0.0
sm40:1	1189.5 ± 159.9	736.6	695.1	705.5
sm40:2	813.9 ± 99.3	570.3	580.3	445.5
sm40:3	44.6 ± 24.5	24.6	25.0	29.0
sm42:0	31.2 ± 15.6	22.4	21.5	64.9
sm42:1	1392.5 ± 176	1,889.8	1,378.2	2,145.0
sm42:2	3640.1 ± 561.8	3,220.9	4,143.8	4,178.4
sm42:3	1399.8 ± 179.2	1,079.4	1,153.3	1,122.9
sm44:1	7.9 ± 1.4	73.8	12.7	62.0
sm44:2	55.7 ± 12.6	257.8	68.9	119.5

[a]pmol/mg protein, n.q. below the quantitation range
*$p < 0.05$ for KO (#1, #2 and #3)/wild-type > 1 (Mann-Whitney U test)
