# Supplementary material for: Hexacosenoyl-CoA is the most abundant very long-chain acyl-CoA in ATP binding cassette transporter D1-deficient cells
Source: J Lipid Res. 2020 Feb 19;61(4):523–36. doi: 10.1194/jlr.P119000325 (PMC7112142; doi:10.1194/jlr.P119000325)
Supplement: Supplemental Data [file supp_P119000325_155112_2_supp_476154_q5pyyv.pdf]

Supplemental Table S1 The quantity of each PL species in the X-ALD fibroblasts

| Signals | CCALD ($n$ = 4)[a] | AMN ($n$ = 3)[a] | Control ($n$ = 5)[a] |
|---|---|---|---|
| pc32:0 | 5625.7 ± 734.6[*,#] | 3939.3 ± 337.3[*] | 3741.1 ± 227.8 |
| pc32:1 | 6962.3 ± 1300.6 | 5813.5 ± 831.5 | 4834.7 ± 427.0 |
| pc32:2 | 882.8 ± 263.7 | 766.6 ± 36.9[*] | 639.8 ± 59.2 |
| pc34:0 | 1934.1 ± 242.3 | 2986.9 ± 1811.8 | 2453.6 ± 1280.1 |
| pc34:1 | 10675.1 ± 1187.6[**] | 8288.8 ± 1377.7 | 6468.6 ± 754.3 |
| pc34:2 | 6705.9 ± 1480.2 | 6126.8 ± 1095.2 | 4999.8 ± 337.8 |
| pc34:3 | 871.8 ± 271.3 | 885.7 ± 87.0 | 670.9 ± 53.3 |
| pc34:4 | 452.1 ± 212.0 | 491.9 ± 85.7 | 303.4 ± 43.4 |
| pc34:5 | 21.4 ± 10.9 | 21.9 ± 10.1 | 13.1 ± 2.9 |
| pc36:0 | 109.5 ± 28.4 | 337.4 ± 415.5 | 207.4 ± 253.6 |
| pc36:1 | 8808.1 ± 1408.4[*] | 8008.9 ± 1851.7 | 5762.1 ± 764.6 |
| pc36:2 | 9817.8 ± 1775.2 | 8421.2 ± 2036.4 | 6707.7 ± 646.7 |
| pc36:3 | 4536.6 ± 843.2 | 4624.1 ± 768.7 | 3612.0 ± 280.3 |
| pc36:4 | 4751.4 ± 768.2[*] | 4246.4 ± 623.3 | 3211.8 ± 409.1 |
| pc36:5 | 1150.1 ± 373.1 | 1209.0 ± 301.3 | 863.2 ± 178.8 |
| pc36:6 | 80.8 ± 29.9 | 85.9 ± 30.6 | 58.3 ± 11.5 |
| pc38:0 | 24.1 ± 2.7[#] | 36.2 ± 2.5 | 28.9 ± 4.8 |
| pc38:1 | 182.1 ± 12.2 | 236.9 ± 78.4 | 187.9 ± 20.9 |
| pc38:2 | 416.7 ± 145.6 | 496.0 ± 170.3 | 279.2 ± 23.6 |
| pc38:3 | 1624.0 ± 181.0 | 2514.7 ± 1028.4 | 1440.8 ± 341.7 |
| pc38:4 | 5507.1 ± 671.0[*] | 4998.0 ± 803.5 | 3811.5 ± 412.4 |
| pc38:5 | 5913.5 ± 1136.4 | 5388.5 ± 1185.8 | 4034.8 ± 530.7 |
| pc38:6 | 1514.1 ± 453.5 | 1605.3 ± 442.9 | 1086.4 ± 241.7 |
| pc38:7 | 181.4 ± 63.2 | 188.5 ± 41.8 | 145.7 ± 35.9 |
| pc38:8 | 7.4 ± 3.8 | 9.0 ± 2.1 | 4.4 ± 0.7 |
| pc40:0 | 13.6 ± 2.1 | 20.6 ± 6.7 | 6.3 ± 0.7 |
| pc40:1 | 27.8 ± 2.1[*] | 41.5 ± 16.5 | 17.7 ± 1.5 |
| pc40:2 | 43.7 ± 8.0 | 61.5 ± 30.8 | 32.7 ± 4.6 |
| pc40:3 | 46.7 ± 6.0 | 69.2 ± 20.9 | 42.3 ± 6.2 |
| pc40:4 | 590.1 ± 105.0 | 700.3 ± 27.5 | 649.1 ± 198.9 |
| pc40:5 | 1980.5 ± 184.3 | 2009.5 ± 313.4 | 1634.6 ± 364.1 |
| pc40:6 | 1013.6 ± 345.5 | 891.9 ± 127.4 | 692.0 ± 115.2 |
| pc40:7 | 729.5 ± 304.7 | 634.2 ± 156.7 | 427.1 ± 75.9 |
| pc40:8 | 147.2 ± 65.1 | 163.1 ± 65.6 | 86.6 ± 12.3 |
| pc40:9 | 15.9 ± 7.4 | 19.6 ± 10.7 | 8.6 ± 1.5 |
| pc40:10 | 1.2 ± 1.7 | 1.6 ± 1.5 | 0.8 ± 0.5 |
| pc42:0 | 18.6 ± 3.7 | 32.1 ± 16.0 | 1.8 ± 0.3 |
| pc42:1 | 35.9 ± 5.7[**] | 58.4 ± 35.4 | 12.1 ± 3.1 |
| pc42:2 | 28.7 ± 4.9[*] | 44.7 ± 27.0 | 13.6 ± 3.9 |
| pc42:3 | 20.6 ± 3.7[*] | 28.6 ± 13.0 | 13.1 ± 2.6 |
| pc42:4 | 38.8 ± 8.0 | 44.3 ± 9.7 | 48.3 ± 14.6 |
| pc42:5 | 129.2 ± 23.3 | 127.7 ± 25.1 | 134.9 ± 35.5 |
| pc42:6 | 110.5 ± 21.9 | 103.2 ± 20.6 | 102.6 ± 20.3 |
| pc42:7 | 106.9 ± 34.9 | 99.2 ± 10.8 | 77.6 ± 14.6 |
| pc42:8 | 113.1 ± 45.1 | 89.2 ± 5.4 | 68.1 ± 12.9 |
| pc42:9 | 251.8 ± 124.2 | 213.3 ± 59.4 | 128.6 ± 27.6 |
| pc42:10 | 121.0 ± 48.9 | 122.3 ± 62.8 | 68.7 ± 12.9 |
| pc42:11 | 4.7 ± 1.8 | 5.9 ± 4.5 | 2.7 ± 1.0 |
| pc44:0 | 6.1 ± 1.5 | 9.9 ± 4.7 | n.q. |
| pc44:1 | 29.5 ± 12.4[*] | 45.0 ± 32.6 | 1.4 ± 0.9 |
| pc44:2 | 23.2 ± 6.8[*] | 37.7 ± 25.4 | 3.0 ± 0.9 |
| pc44:3 | 9.8 ± 2.3 | 13.4 ± 8.1 | 2.9 ± 1.1 |
| pc44:4 | 9.8 ± 3.4 | 11.0 ± 4.6 | 5.0 ± 1.8 |
| pc44:5 | 19.4 ± 5.0 | 19.3 ± 6.7 | 10.8 ± 3.3 |
| pc44:6 | 15.8 ± 5.4 | 17.2 ± 7.4 | 14.4 ± 4.4 |
| pc44:7 | 24.5 ± 10.3 | 25.0 ± 7.1 | 21.7 ± 6.2 |
| pc44:8 | 30.1 ± 11.0 | 23.1 ± 4.6 | 27.7 ± 8.0 |
| pc44:9 | 49.2 ± 15.8 | 39.6 ± 3.2 | 41.3 ± 8.1 |
| pc44:10 | 52.0 ± 21.7 | 45.4 ± 8.6 | 31.3 ± 6.0 |
| pc44:11 | 59.9 ± 33.5 | 47.2 ± 17 | 26.8 ± 7.2 |
| pc44:12 | 9.1 ± 4.0 | 7.1 ± 3.5 | 4.6 ± 1.4 |
| pc46:0 | n.q. | 1.1 ± 1.1 | n.q. |
| pc46:1 | 4.7 ± 2.5 | 6.0 ± 4.3 | n.q. |
| pc46:2 | 4.2 ± 2.1 | 5.3 ± 3.3 | n.q. |
| pc46:3 | 3.5 ± 1.0 | 3.5 ± 1.6 | n.q. |
| pc46:4 | 12.9 ± 4.6 | 12.2 ± 7.7 | 0.2 ± 0.5 |

| Signals | CCALD ($n$ = 4)[a] | AMN ($n$ = 3)[a] | Control ($n$ = 5)[a] |
|---|---|---|---|
| pc46:5 | 14.4 ± 4.0 | 14.3 ± 6.9 | 1.9 ± 0.5 |
| pc46:6 | 7.3 ± 2.0 | 8.4 ± 4.6 | 2.7 ± 1.1 |
| pc46:7 | 2.6 ± 1.9 | 3.0 ± 1.3 | 2.3 ± 1.3 |
| pc46:8 | 8.4 ± 3.6 | 7.0 ± 3.1 | 9.5 ± 4.4 |
| pc46:9 | 7.2 ± 3.2 | 4.6 ± 0.7 | 6.5 ± 2.4 |
| pc46:10 | 7.9 ± 3.1 | 6.1 ± 2.0 | 7.4 ± 2.9 |
| pc46:11 | 2.4 ± 1.9 | 2.0 ± 0.6 | 1.9 ± 0.7 |
| pc48:4 | 2.0 ± 1.9 | 1.5 ± 1.5 | n.q. |
| pc48:5 | 5.6 ± 2.2 | 5.2 ± 2.9 | n.q. |
| pc48:6 | 1.5 ± 1.1 | 1.9 ± 0.7 | n.q. |
| pc48:10 | 1.2 ± 1.4 | 0.8 ± 0.8 | 1.3 ± 0.8 |
| pe32:0 | 382.3 ± 131.4 | 253.6 ± 47.6 | 355.1 ± 170.2 |
| pe32:1 | 860.5 ± 181.8 | 911.0 ± 112.0 | 828.1 ± 175.0 |
| pe32:2 | 72.5 ± 20.0 | 84.5 ± 10.0 | 69.5 ± 13.8 |
| pe34:0 | 556.4 ± 170.7 | 539.5 ± 82.5 | 628.6 ± 214.4 |
| pe34:1 | 8837.3 ± 1774.1 | 11493.7 ± 3386 | 8192.4 ± 1358.2 |
| pe34:2 | 1917.4 ± 460.0 | 2419.7 ± 421.9 | 1782.2 ± 237.2 |
| pe34:4 | 83.4 ± 28.6 | 106.0 ± 13.6[*] | 67 ± 12.4 |
| pe34:5 | 2.3 ± 1.2 | 3.5 ± 1.6 | 2.0 ± 0.7 |
| pe36:0 | 29.6 ± 7.3 | 37.4 ± 10.9 | 31.6 ± 10.8 |
| pe36:1 | 13922.4 ± 2513.5 | 18709.6 ± 3210.3 | 12814.5 ± 2693.5 |
| pe36:2 | 8602.3 ± 1458.7 | 12374.0 ± 3173.9 | 8199.3 ± 876.6 |
| pe36:3 | 1755.8 ± 317.3 | 2619.7 ± 481.7 | 1799.6 ± 253.1 |
| pe36:4 | 1682.4 ± 349.1 | 2249.5 ± 626.5 | 1622.9 ± 229.9 |
| pe36:5 | 229.7 ± 76.3 | 321.7 ± 109.4 | 223.3 ± 51.8 |
| pe36:6 | 38.4 ± 9.7 | 53.8 ± 12.5 | 43.6 ± 6.3 |
| pe36:7 | n.q. | 1.9 ± 0.8 | 0.8 ± 0.7 |
| pe38:0 | 3.1 ± 0.7 | 3.8 ± 2.0 | 2.9 ± 1.0 |
| pe38:1 | 331.4 ± 43.5 | 408.3 ± 64.5 | 322.3 ± 68.0 |
| pe38:2 | 344.4 ± 64.5 | 558.4 ± 112.2 | 351.8 ± 61.3 |
| pe38:3 | 2318.7 ± 675.3 | 4483.6 ± 1380.2 | 2873.6 ± 833.4 |
| pe38:4 | 12768.6 ± 1304.7 | 16222.0 ± 4100.8 | 12409.2 ± 500.2 |
| pe38:5 | 6158.7 ± 1271.9 | 8561.6 ± 2276.5 | 5741.9 ± 488.8 |
| pe38:6 | 2474.6 ± 693.8 | 2885.2 ± 636.2 | 2238.5 ± 513.3 |
| pe38:7 | 483.8 ± 238.2 | 422.8 ± 68.2 | 323.2 ± 43.7 |
| pe38:8 | 2.0 ± 2.2 | 2.1 ± 2.8 | 2.7 ± 3.1 |
| pe40:0 | 3.4 ± 1.0 | 3.5 ± 1.6 | 1.8 ± 1.1 |
| pe40:1 | 142.2 ± 50.1 | 159.5 ± 69.3 | 157.9 ± 37.7 |
| pe40:2 | 184.6 ± 45.0 | 213.0 ± 46.7 | 186.2 ± 50.2 |
| pe40:3 | 86.3 ± 19.7 | 166.2 ± 18.2[*] | 101.4 ± 34.7 |
| pe40:4 | 4144.6 ± 1443.9 | 4581.9 ± 840.1 | 3959.6 ± 1555.3 |
| pe40:5 | 6328.9 ± 1758.0 | 6925.2 ± 656.5 | 6111.2 ± 2206.2 |
| pe40:6 | 4781.3 ± 1493.2 | 3840.2 ± 770.6 | 3401.8 ± 1059.9 |
| pe40:7 | 2462.9 ± 744.2 | 2508.0 ± 489.0 | 1961.9 ± 540.7 |
| pe40:8 | 157.0 ± 86.6 | 194.2 ± 47.4 | 101.4 ± 23.4 |
| pe40:9 | 18.8 ± 13.8 | 25.3 ± 9.0 | 10.8 ± 3.2 |
| pe40:10 | 1.8 ± 2.5 | 2.7 ± 1.0 | 1.0 ± 1.0 |
| pe42:0 | 0.6 ± 0.7 | 2.2 ± 1.9 | n.q. |
| pe42:1 | 159.6 ± 47.4 | 186.2 ± 113.4 | 108.9 ± 34.4 |
| pe42:2 | 117.4 ± 26.1 | 120.7 ± 41.7 | 83.6 ± 29.1 |
| pe42:3 | 42.9 ± 12.9 | 50.5 ± 19.0 | 38.1 ± 12.5 |
| pe42:4 | 48.5 ± 20.0 | 70.2 ± 13.4 | 77.5 ± 36.0 |
| pe42:5 | 172.2 ± 45.5 | 219.0 ± 15.9 | 210.6 ± 60.4 |
| pe42:6 | 113.9 ± 33.0 | 163.1 ± 13.5 | 125.5 ± 38.4 |
| pe42:7 | 143.1 ± 41.6 | 172.6 ± 22.5 | 123.9 ± 41.9 |
| pe42:8 | 88.6 ± 22.7 | 138.2 ± 1.2 | 80.7 ± 26.3 |
| pe42:9 | 33.6 ± 13.9 | 51.6 ± 8.1 | 28.5 ± 6.8 |
| pe42:10 | 45.9 ± 21.8 | 56.3 ± 14.6 | 34.2 ± 7.9 |
| pe42:11 | 1.3 ± 2.1 | 1.7 ± 1.5 | 1.0 ± 0.6 |
| pe44:1 | 54.6 ± 23.5 | 61.2 ± 36.7 | 5.1 ± 1.5 |
| pe44:2 | 30.4 ± 13.0 | 33.3 ± 16.2 | 6.0 ± 2.1 |
| pe44:3 | 13.3 ± 5.3 | 16.0 ± 7.9 | 7.3 ± 2.5 |
| pe44:4 | 7.0 ± 5.2 | 7.5 ± 2.9 | 6.4 ± 4.5 |
| pe44:5 | 34.2 ± 14.0 | 41.1 ± 21.2 | 27.5 ± 14.4 |

| Signals | CCALD ($n$ = 4)[a] | AMN ($n$ = 3)[a] | Control ($n$ = 5)[a] |
|---|---|---|---|
| pe44:6 | 14.1 ± 7.1 | 12.1 ± 3.1 | 18.0 ± 9.6 |
| pe44:7 | 12.7 ± 6.0 | 3.1 ± 2.8 | 8.0 ± 6.2 |
| pe44:8 | 13.6 ± 5.3 | 16.4 ± 6.3 | 16.2 ± 5.4 |
| pe44:9 | 17.0 ± 6.3 | 24.8 ± 4.3 | 21.4 ± 3.7 |
| pe44:10 | 11.0 ± 4.7 | 15.7 ± 0.8 | 10.6 ± 3.1 |
| pe44:11 | 5.6 ± 3.1 | 9.8 ± 2.5 | 6.1 ± 1.8 |
| pe44:12 | 2.7 ± 1.8 | 5.4 ± 2.6 | 3.1 ± 0.8 |
| pe46:1 | 0.2 ± 0.4 | 1.6 ± 1.4 | n.q. |
| pe46:2 | 0.6 ± 1.2 | 1.4 ± 1.3 | n.q. |
| pe46:3 | n.q. | 1.6 ± 1.6 | n.q. |
| pe46:5 | 14.1 ± 7.7 | 14.8 ± 3.8 | 7.0 ± 5.1 |
| pe46:6 | 12.8 ± 6.8 | 9.3 ± 0.4 | 7.1 ± 5.7 |
| pe46:7 | 8.8 ± 5.4 | 5.6 ± 1.5 | 4.2 ± 2.7 |
| pe46:10 | 1.6 ± 1.2 | 2.4 ± 0.3 | 3.1 ± 1.4 |
| pe46:11 | 1.2 ± 1.9 | 0.7 ± 1.2 | 1.6 ± 0.9 |
| pe48:5 | 2.9 ± 2.1 | 1.3 ± 1.1 | n.q. |
| pe48:6 | 1.3 ± 1.5 | 1.1 ± 0.9 | n.q. |
| sm32:0 | 373.0 ± 30.7 | 423.6 ± 195.1 | 360.5 ± 43.9 |
| sm32:1 | 1503.9 ± 226.5 | 1563.2 ± 80.7** | 1135.2 ± 111.3 |
| sm32:2 | 31.8 ± 5.7 | 40.1 ± 6.7 | 24.5 ± 5.2 |
| sm34:0 | 4753.4 ± 1001.0 | 3196.5 ± 2545.1 | 4010.7 ± 594.0 |
| sm34:1 | 20207.5 ± 2496.3** | 13219.1 ± 2755.5 | 11768.5 ± 1048.5 |
| sm34:2 | 2294.6 ± 202.9 | 3138.9 ± 387.8* | 2098.5 ± 412.7 |
| sm36:0 | 206.8 ± 41.4 | 161.5 ± 138.5 | 225.7 ± 53.5 |
| sm36:1 | 2064.6 ± 494.6 | 1959.6 ± 812.6 | 2029.6 ± 186.2 |
| sm36:2 | 517.4 ± 190.9 | 635.5 ± 248.1 | 424.9 ± 73.0 |
| sm36:3 | 32.0 ± 8.8 | 35.2 ± 9.4 | 25.8 ± 5.2 |
| sm38:0 | 61.6 ± 9.7 | 80.0 ± 14.2 | 75.5 ± 13.2 |
| sm38:1 | 366.4 ± 66.5* | 579.1 ± 347.9 | 547.3 ± 99.5 |
| sm38:2 | 48.3 ± 5.6 | 85.5 ± 63.2 | 55.2 ± 12.5 |
| sm38:3 | 31.9 ± 7.4 | 23.9 ± 3.0 | 19.7 ± 4.5 |
| sm40:0 | 161.7 ± 62.6 | 96.6 ± 91.6 | 173.8 ± 55.3 |
| sm40:1 | 2496.2 ± 625.8 | 2460.8 ± 449.4 | 3494.5 ± 600.4 |
| sm40:2 | 1044.5 ± 175.3 | 1407.9 ± 389.6 | 1347.2 ± 223.1 |
| sm40:3 | 7.9 ± 15.8 | 38.5 ± 66.8 | 15.7 ± 35.0 |
| sm42:0 | 78.6 ± 14.7 | 71.6 ± 70.6 | 78.8 ± 16.9 |
| sm42:1 | 4925.8 ± 1227.5 | 4577.2 ± 655.4 | 5027.6 ± 852.6 |
| sm42:2 | 9785.3 ± 2823.5 | 10046.7 ± 1290.7 | 9760.5 ± 944.0 |
| sm42:3 | 2819.7 ± 772.5 | 2839.5 ± 412.3 | 3072.2 ± 370.2 |
| sm44:1 | 248.2 ± 77.4* | 193.3 ± 21.8** | 42.0 ± 9.0 |
| sm44:2 | 420.6 ± 129.4 | 355.6 ± 37.9* | 153.6 ± 22.8 |
| sm44:3 | 258.3 ± 86.7 | 232.1 ± 30.8* | 136.2 ± 18.9 |
| sm46:1 | 1.4 ± 1.2 | 0.6 ± 1 | n.q. |
| sm46:2 | 2.6 ± 1.5 | 2.0 ± 0.8 | n.q. |
| sm46:3 | 2.1 ± 1.6 | 2.1 ± 0.5 | 0.4 ± 0.5 |

[a]pmol/mg protein, n.q. below the quantitation range
*$p < 0.05$, **$p < 0.01$ vs. control (Dunnett T3 post hoc test)

#$p < 0.05$ vs. AMN (Dunnett T3 post hoc test)
