# Supplementary material for: Hexacosenoyl-CoA is the most abundant very long-chain acyl-CoA in ATP binding cassette transporter D1-deficient cells
Source: J Lipid Res. 2020 Feb 19;61(4):523–36. doi: 10.1194/jlr.P119000325 (PMC7112142; doi:10.1194/jlr.P119000325)
Supplement: Supplemental Data [file supp_P119000325_155112_2_supp_476155_q5pyyv.pdf]

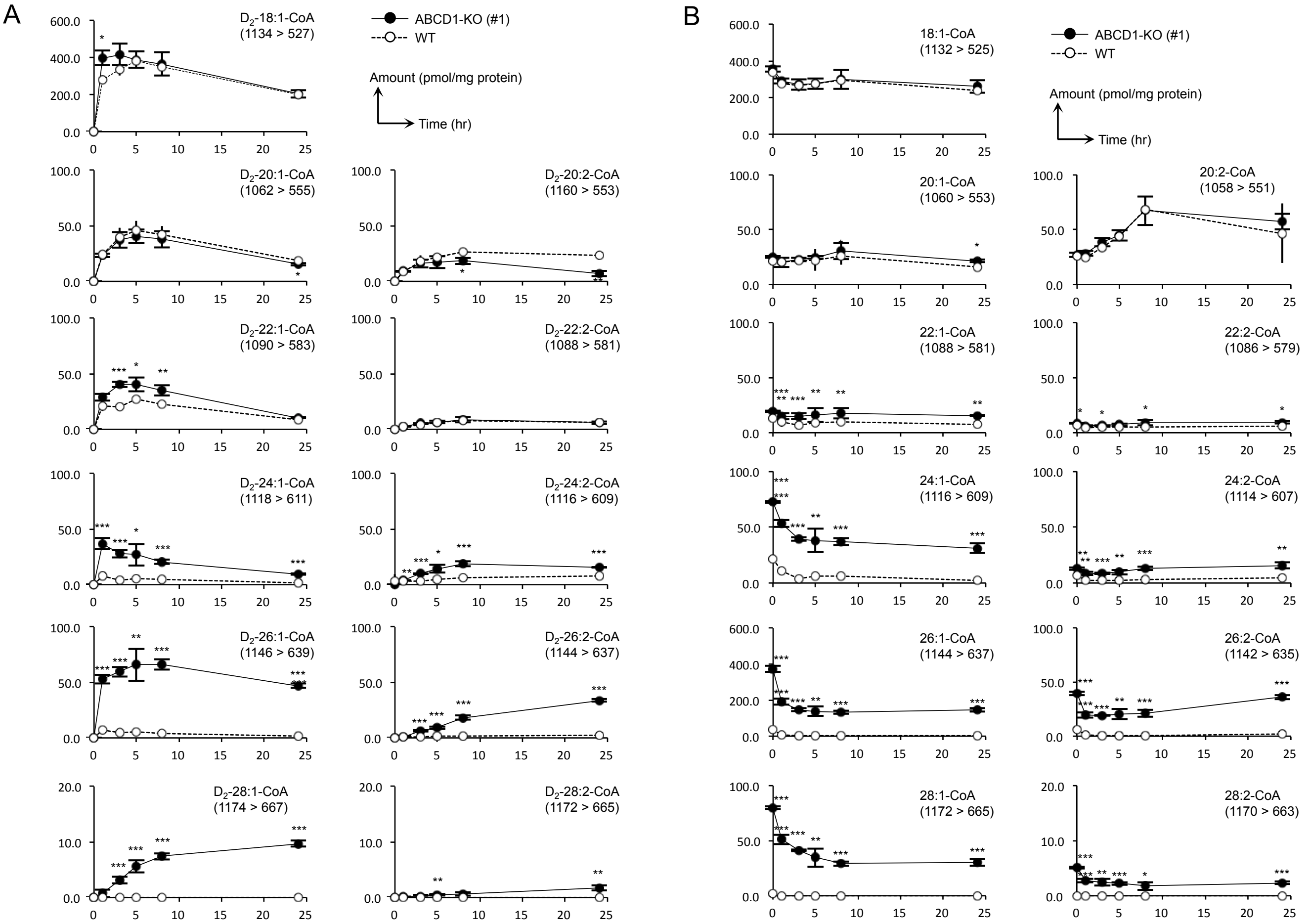

Supplemental Figure S3. Metabolic analysis of VLCFA-CoA species using FA D_2-18:1. Metabolic profiles of each labeled or non-labeled acyl-CoA species were examined in ABCD1-KO (#1) and wild-type (WT) HeLa cells. Deuterium-labeled acyl-CoA species (A) and non-labeled acyl-CoA species (B) are represented. Note that the four panels for D_2-18:1-, D_2-26:1-, 18:1-, and 26:1-CoA are identical with those shown in Fig. 5. Statistical analyses were performed with Student's *t*-test; *$p < 0.05$, **$p < 0.01$, ***$p < 0.001$
