# Supplementary material for: Hexacosenoyl-CoA is the most abundant very long-chain acyl-CoA in ATP binding cassette transporter D1-deficient cells
Source: J Lipid Res. 2020 Feb 19;61(4):523–36. doi: 10.1194/jlr.P119000325 (PMC7112142; doi:10.1194/jlr.P119000325)
Supplement: Supplemental Data [file supp_P119000325_155112_2_supp_476156_q5pyyv.pdf]

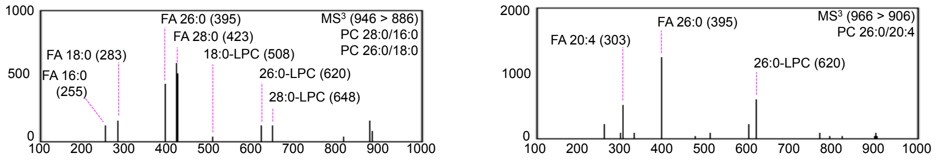

Supplemental Figure S2. The product ion spectra of VLCFA-PL species present in quantities significantly higher in the ABCD1-KO HeLa cells. The ions corresponding to $[M+HCOO]^-$ and $[M-CH_3]^-$ were selected as the first and second precursor ions, respectively in LC-MS^3 analysis
